# Next-generation proteomics of serum extracellular vesicles combined with single-cell RNA sequencing identifies MACROH2A1 associated with refractory COVID-19

**DOI:** 10.1186/s41232-022-00243-5

**Published:** 2022-11-30

**Authors:** Takahiro Kawasaki, Yoshito Takeda, Ryuya Edahiro, Yuya Shirai, Mari Nogami-Itoh, Takanori Matsuki, Hiroshi Kida, Takatoshi Enomoto, Reina Hara, Yoshimi Noda, Yuichi Adachi, Takayuki Niitsu, Saori Amiya, Yuta Yamaguchi, Teruaki Murakami, Yasuhiro Kato, Takayoshi Morita, Hanako Yoshimura, Makoto Yamamoto, Daisuke Nakatsubo, Kotaro Miyake, Takayuki Shiroyama, Haruhiko Hirata, Jun Adachi, Yukinori Okada, Atsushi Kumanogoh

**Affiliations:** 1 Department of Respiratory Medicine and Clinical Immunology, Graduate School of Medicine, Osaka University, Osaka, 565-0871 Japan; 2 Laboratory of Immunopathology, World Premier International Immunology Frontier Research Center (WPI-IFReC), Osaka University, Osaka, 565-0871 Japan; 3 Department of Statistical Genetics, Osaka University Graduate School of Medicine, Suita, Japan; 4 Laboratory of Bioinformatics, Artificial Intelligence Center for Health and Biomedical Research, National Institutes of Biomedical Innovation, Health and Nutrition, 7-6-8 Saito-Asagi, Ibaraki, Osaka 567-0085 Japan; 5 Department of Respiratory Medicine, National Hospital Organization Osaka Toneyama Medical Center, 5-1-1 Toneyama, Toyonaka, Osaka 560-8552 Japan; 6 Laboratory of Proteomics for Drug Discovery, Center for Drug Design Research, National Institute of Biomedical Innovation, Health and Nutrition, 7-6-8, Saito-Asagi, Ibaraki City, Osaka, 567-0085 Japan; 7 Center for Infectious Diseases for Education and Research (CiDER), Osaka University, Suita, Osaka Japan; 8 Integrated Frontier Research for Medical Science Division, Institute for Open and Transdisciplinary Research Initiatives (OTRI), Osaka University, Suita, Japan; 9 Japan Agency for Medical Research and Development-Core Research for Evolutionary Medical Science and Technology (AMED-CREST), Japan Agency for Medical Research and Development, Tokyo, Japan; 10 Center for Advanced Modalities and DDS (CAMaD), Osaka University, Osaka, Japan

**Keywords:** COVID-19, SARS-CoV-2, Exosome, MACROH2A1, Multi-omics, Liquid biopsy

## Abstract

**Background:**

The coronavirus disease 2019 (COVID-19) pandemic is widespread; however, accurate predictors of refractory cases have not yet been established. Circulating extracellular vesicles, involved in many pathological processes, are ideal resources for biomarker exploration.

**Methods:**

To identify potential serum biomarkers and examine the proteins associated with the pathogenesis of refractory COVID-19, we conducted high-coverage proteomics on serum extracellular vesicles collected from 12 patients with COVID-19 at different disease severity levels and 4 healthy controls. Furthermore, single-cell RNA sequencing of peripheral blood mononuclear cells collected from 10 patients with COVID-19 and 5 healthy controls was performed.

**Results:**

Among the 3046 extracellular vesicle proteins that were identified, expression of MACROH2A1 was significantly elevated in refractory cases compared to non-refractory cases; moreover, its expression was increased according to disease severity. In single-cell RNA sequencing of peripheral blood mononuclear cells, the expression of *MACROH2A1* was localized to monocytes and elevated in critical cases. Consistently, single-nucleus RNA sequencing of lung tissues revealed that *MACROH2A1* was highly expressed in monocytes and macrophages and was significantly elevated in fatal COVID-19. Moreover, molecular network analysis showed that pathways such as “estrogen signaling pathway,” “p160 steroid receptor coactivator (SRC) signaling pathway,” and “transcriptional regulation by STAT” were enriched in the transcriptome of monocytes in the peripheral blood mononuclear cells and lungs, and they were also commonly enriched in extracellular vesicle proteomics.

**Conclusions:**

Our findings highlight that MACROH2A1 in extracellular vesicles is a potential biomarker of refractory COVID-19 and may reflect the pathogenesis of COVID-19 in monocytes.

**Supplementary Information:**

The online version contains supplementary material available at 10.1186/s41232-022-00243-5.

## Background

The globally widespread severe acute respiratory syndrome coronavirus 2 (SARS-CoV-2) infections are overwhelming medical institutions. Although vaccination is highly effective, the emerging Omicron variant is highly transmissible and vaccine-resistant; thus, leading to a resurgence of coronavirus disease 2019 (COVID-19) [1].

In general, > 80% of patients with COVID-19 recover with mild illness, while it is severe in others [2–4]. Currently, there are no effective predictors, which can predict the patients who may become severely ill, due to highly variable patient responses to SARS-CoV-2 infection. Furthermore, in severe cases, anti-inflammatory treatments, such as corticosteroids, are administered; however, there are refractory cases wherein the condition does not ameliorate even with standard treatment [5–7]. Some studies have reported that laboratory data, such as CRP, D-dimer, and lymphocyte counts, might predict aggravation of COVID-19 pneumonia [8–10]. Although these markers can easily be measured in clinical practice, they are not highly specific in predicting the development of COVID-19 pneumonia; hence, their usefulness is limited. Therefore, a more comprehensive and deeper protein analysis, using easily available peripheral blood samples is necessary to identify a reliable marker of severe and refractory COVID-19 that can be used in clinical practice. However, biomarker candidates that are present in low concentrations in serum may be masked when investigated by conventional proteomics because 99% of serum proteins comprise of proteins such as albumin, complement factors, and immunoglobulins [11].

Extracellular vesicles (EVs) are small, lipid bilayer-enclosed vesicles that contain several bio-molecules, including proteins and microRNAs [12]. EVs are secreted by most cell types and have various physiological functions, such as intercellular communication and immune responses [13]. Since EVs circulate through the body fluids and their cargos are protected by the lipid bilayer membrane, they are considered an ideal biomarker source. Notably, in various diseases, including COVID-19, EVs have been reported to contain molecules of biomarker candidates [14–17]. In addition, data-independent acquisition (DIA), which is a far more comprehensive and reproducible proteomic analysis method than the conventional data-dependent acquisition (DDA), is a recent advancement in proteomics technology [18–20]. Although this “next-generation proteomics” approach-based DIA analysis of EVs is being applied to biomarker discovery research for various diseases [21–23], investigation by this technique has not been satisfactory.

Several studies have performed multi-omics analysis, including scRNA-seq, proteomics, and metabolomics, to better understand the coordinated systemic immune response in patients with severe COVID-19. However, these studies lack high-coverage proteomics and mainly focus on dysregulation of immune cells and proteins that were key molecules in severe COVID-19, and useful biomarkers that predict refractory diseases were not fully investigated. One of these reports that performed single-cell RNA sequencing (scRNA-seq) of peripheral blood mononuclear cells (PBMC) and plasma proteomics and metabolomics, revealed changes in immune response among different COVID-19 severity levels; however, the proteomics identified only 464 proteins [24]. Lipidomics and proteomics of EVs from COVID-19 revealed changes in EV lipid raft metabolism between different stages of the disease; however, its proteomics by DDA identified only a total of 142 proteins [25]. Although these studies highlight a part of the pathophysiology of COVID-19, the analyses were insufficient to identify specific biomarker proteins. Herein, we have integrated next-generation proteomics and single-cell transcriptomics of peripheral blood to find biomarker candidates of severe SARS-CoV-2 infection and successfully identified a novel key molecule, MACROH2A1*.*

## Methods

### Study design

For proteomic analysis, 12 patients with COVID-19 and 4 healthy controls were enrolled. COVID-19 severity on admission was categorized as non-critical or critical based on the “Living guidance for clinical management of COVID-19” (WHO, 2021). Briefly, “critical” patients are those who required life-sustaining treatment, and “non-critical” patients are those who were not “critical” patients. In this study, all “critical” cases were under mechanical ventilation and all “non-critical patients” were not. Patients with COVID-19 were diagnosed by polymerase chain reaction tests for SARS-CoV-2 RNA; critical and non-critical patients comprised those admitted to Osaka University Hospital and National Hospital Organization, Toneyama Hospital, respectively, between July 2020 and February 2021. Healthy controls were recruited at Osaka University Hospital in the pre-COVID-19 era. All patients with COVID-19 were treated with dexamethasone based on RECOVERY trial [6], and serum samples were collected at the end of the dexamethasone treatment and stored at − 80 °C. Medical records of these patients were retrospectively analyzed. In this study, we categorized the critical cases in which COVID-19 pneumonia worsened in the subsequent course and required re-administration of dexamethasone as refractory cases, and the remaining critical cases in which dexamethasone was not required were classified as non-refractory cases. The decision to re-administer dexamethasone was taken by the respective physicians when all of the following persisted after other infections had been ruled out [26, 27]: (1) worsening respiratory status, (2) worsening radiographical images indicating pneumonia, (3) elevated systemic inflammatory markers such as serum C-reactive protein or fever. We used these samples to conduct quantitative proteomics using liquid chromatography-mass spectrometry (LC–MS/MS). For scRNA-seq analysis, all patients with COVID-19 were admitted to Osaka University Hospital. COVID-19 severity and diagnosis were determined similarly to how they were determined during the proteomic analysis. Critical cases comprised the same patients whose samples were used in the proteomic analysis.

### Isolation of EVs for proteomics

EVs were isolated using MagCapture™ Exosome Isolation Kit PS Ver.2 (FUJIFILM Wako, Japan), according to the manufacturer’s instruction. Subsequently, serum samples were boiled at 80 °C for 10 min to inactivate the SARS-CoV-2 virus. Size distributions and numbers of the EVs were measured by NanoSight nanoparticle tracking analysis (Malvern Instruments, Malvern, UK).

### Proteomic analysis

Proteomic analysis of serum EVs was performed as described in a previous study [14]. The exosome eluate was boiled at 95 °C for 5 min after adding 120 mM sodium deoxycholate, 10 × phase-transfer surfactant (PTS) buffer comprising 500 mM NH_4_HCO_3_, and 120 mM sodium N-lauroyl sarcosinate. The sample was kept at 37 °C for 30 min after adding 10 mM TCEP; subsequently, 20 mM iodoacetamide was added to it and it was kept at 37 °C for 30 min in the dark for alkylation, followed by overnight digestion at 37 °C with 1 μg trypsin (Wako-Chemical, Tokyo, Japan) and 2 mAU LysC (Wako-Chemical, Tokyo, Japan). One percent trifluoroacetic acid (TFA) was added to the digested solutions, and the detergents were precipitated by centrifugation at 20,000* g* for 10 min. The supernatant containing the fragmented peptide was desalted by adding a C18-SCX StageTip and further dried with a centrifugal evaporator. LC–MS/MS was conducted by coupling an HTC-PAL autosampler (CTC Analytics, Zwingen, Switzerland) and an UltiMate 3000 Nano LC system (Thermo Scientific, Bremen, Germany) to an Orbitrap Fusion Lumos mass spectrometer (Thermo Scientific). Peptides were separated at a flow rate of 280 nL/min using a 45-min gradient from 5 to 30% of solvent B (solvent A, 0.1% formic acid (FA); solvent B, 0.1% FA and 99.9% acetonitrile) at an analytical column (75 μm × 20 cm, packed in-house with ReproSil-Pur C18-AQ, 1.9 μm resin, Dr. Maisch, Ammerbuch, Germany). Operation of the Orbitrap Fusion Lumos mass spectrometer was performed under the 5 GPF (gas-phase fractionation)-DIA mode (50,000 fragment resolution, 120,000 precursor resolution, automatic gain control (AGC) target of 1e6 and 2e5 for MS1 and MS2, max IIT of 250 ms and 86 ms for MS1 and MS2, NCE of 30, and 2 m/z precursor isolation window), and it covered 418–494, 490–566, 562–638, 634–710, and 706–782 m/z (5xGPF). Analysis of individual samples was conducted in the DIA mode (30,000 fragment resolution, 120,000 precursor resolution, AGC target of 4e5 and 2e5 for MS1 and MS2, max IIT of 100 ms, and 54 ms for MS1 and MS2, NCE of 30, and 8 m/z precursor isolation window). Analysis of the DIA data was performed by DIA-NN (version 1.7.12) using the following default settings: scan window setting and automatic mass accuracy tolerance. Search results were qualified and they were filtered to a 1% precursor level. MS files were searched using UniProt human database.

### Bioinformatics analysis of the proteome

To analyze and elucidate biologically relevant proteomic pathways and molecular networks, we implemented the following tools: Ingenuity Pathways Analysis (IPA, Qiagen. Inc. Redwood City, CA, USA) for upstream and enrichment analyses.

### Analysis of molecular networks and pathways for the proteome and transcriptome

KeyMolnet (viewer program version 6.2, contents version 9.7.20210930154837, KM Data Inc) was used for the analysis of molecular networks and pathways for EV proteomics, scRNA-seq of the PBMCs, and snRNA-seq of the lungs as described previously [28]. Briefly, KeyMolnet, a commercial knowledge base, includes core content from reliable selection of a review article and secondary content from important original articles of major journals. It contains about 260,000 relationships among human genes, proteins, microRNAs, and small molecules. Molecular network analysis with the “start points and end-points” network search algorithm was performed to evaluate the molecular network through which deferentially expressed molecules affect the target molecules (analysis of the upstream networks of the target molecules) or, conversely, through which molecular network, the target molecules affect these molecules (analysis of the downstream networks of the target molecules). While analyzing the pathways related to the obtained molecular network, the significance of the similarity between the extracted network and the canonical pathway was scored as HScore using the calculation formula based on the hypergeometric distribution. An HScore of greater than 20 was considered statistically significant [29].

### Cell culture and stimulation

THP-1 was cultured in RPMI medium and stimulated with PMA 5 ng/mL for 48 h. Subsequently, the cells were treated with LPS, Pam3CSK4, R848, or interferon (IFN)-gamma. After 48 h, the cell lysate was collected.

### Transmission electron microscopy

EVs from serum samples were adsorbed on nickel grid coated by formvar and carbon and fixed with 2% paraformaldehyde. These samples were incubated with anti-CD9 (MM2/57; Thermo Fisher Scientific).

### Subjects and specimen collection of PBMCs for scRNA-seq

Peripheral blood samples were collected from COVID-19 patients (*n* = 10) and healthy controls (*n* = 5) at Osaka University Hospital and Osaka University Graduate School of Medicine. For both patients with COVID-19 and healthy controls, blood was collected into heparin tubes, and PBMCs were isolated using Leucosep (Greiner Bio-One) density gradient centrifugation according to the manufacturer’s instructions. Blood was processed within 3 h of collection for all samples and stored at − 80 °C until use.

### Droplet-based single-cell sequencing

Single-cell suspensions were processed through the 10 × Genomics Chromium Controller (10 × Genomics, USA) following the protocol outlined in the Chromium Single Cell V(D)J Reagent Kits (v1.1 Chemistry) User Guide. Chromium Next GEM Single Cell 5’ Library & Gel Bead Kit v1.1 (Cat# PN-1000167), Chromium Next GEM Chip G Single Cell Kit (Cat# PN-1000127), and Single Index Kit T Set A (Cat# PN-1000213) were applied during the process. Approximately 16,500 live cells per sample were separately loaded into each port of the 10 × Genomics Chromium controller without sample mixing to generate 10,000 single-cell gel-bead emulsions for library preparation and sequencing, according to the manufacturer’s recommendations. Oil droplets of encapsulated single cells and barcoded beads (GEMs) were subsequently reverse-transcribed in a Veriti Thermal Cycler (Thermo Fisher Scientific), resulting in cDNA tagged with a cell barcode and unique molecular index (UMI). Next, cDNA was amplified to generate single-cell libraries according to the manufacturer’s protocol. Quantification was made with an Agilent Bioanalyzer High-Sensitivity DNA assay (Agilent, High-Sensitivity DNA Kit, Cat# 5067–4626). Subsequently, amplified cDNA was enzymatically fragmented, end-repaired, and polyA tagged. Cleanup/size selection was performed on amplified cDNA using SPRIselect magnetic beads (Beckman-Coulter, SPRIselect, Cat# B23317). Next, Illumina sequencing adapters were ligated to the size-selected fragments and cleaned up using SPRIselect magnetic beads. Finally, sample indices were selected and amplified, followed by a double-sided size selection using SPRIselect magnetic beads. Final library quality was assessed using an Agilent Bioanalyzer High-Sensitivity DNA assay. Samples were then sequenced on NovaSeq6000 (Illumina, USA) as a paired-end mode to achieve a minimum of 20,000 paired-end reads per cell for gene expression.

### Alignment, quantification, and quality control of scRNA-seq data

Droplet libraries were processed using Cell Ranger 5.0.0 (10 × Genomics, USA). Sequencing reads were aligned with STAR [30] using the GRCh38 human reference genome. Count matrices were built from the resulting BAM files using dropEst [31]. Cells that had < 1000 UMIs or > 20,000 UMIs, as well as cells that contained > 10% of reads from mitochondrial genes or hemoglobin genes, were considered low quality and removed from further analysis. Additionally, putative doublets were removed using Scrublet (v0.2.1) for each sample [32].

### scRNA-seq computational pipelines and analysis

The R package Seurat (v3.2.2) was used for data scaling, transformation, clustering, dimensionality reduction, differential expression analysis, and most visualization [33]. Data were scaled and transformed using the SCTransform() function, and linear regression was performed to remove unwanted variation due to cell quality (% mitochondrial reads). For integration, we identified 3000 shared highly variable genes (HVGs) using SelectIntegrationFeatures() function. Then, we identified “anchors” between individual datasets based on these genes using the FindIntegrationAnchors() function and inputted these anchors into the IntegrateData() function to create a batch-corrected expression matrix of all cells. Principal component analysis (PCA) and uniform manifold approximation and projection (UMAP) dimension reduction with 30 principal components were performed [34]. A nearest-neighbor graph using the 30 dimensions of the PCA reduction was calculated using FindNeighbors() function, followed by clustering using FindClusters() function.

Cellular identity was determined by finding differentially expressed genes (DEGs) for each cluster using FindMarkers() function with the parameter “test.use = wilcox” and comparing those markers to known cell type-specific genes (Suppl Fig. 5). We obtained nine cell clusters (Fig. 3b). To clarify immune cell type-specific expression of *MACROH2A1*, we produced the density plot using plot_density() function from Nebulosa R package (v1.0.0) [35], and the dot plot using DotPlot() function. We performed differential expression analysis of *MACROH2A1* between four pairs of comparisons: Group 3 and Group 2, Groups 2 and 3 and Group 1, Groups 2 and 3 and healthy controls, and overall COVID-19 patient groups and healthy controls in each cell type using FindMarkers() function with parameter “test.use = wilcox.”

Droplets labeled as monocytes were extracted and reintegrated for further subclustering using the same procedure as described above. After integration, clustering, cluster annotation (Suppl Fig. 5), and differential expression analysis were performed as described above.

### Analysis of snRNA-seq datasets of COVID-19 lungs

We used publicly available datasets for the analysis of single-cell transcriptome data of COVID-19 lung tissues [36]: Melms et al. performed single-nucleus RNA sequencing (snRNA-seq) of 116,314 nuclei from the lungs of 19 patients with COVID-19 pneumonia who underwent rapid autopsy and seven controls who underwent lung resection or biopsy prior to the COVID-19 pandemic. The expression levels of *MACROH2A1* were examined separately in the COVID-19 and control lungs. The significance of the different expression levels of *MACROH2A1* was assessed using a Wilcoxon rank-sum test. The R package Seurat (v4.1.1) was used in snRNA data analysis.

### Western blotting

Cultured cells were lysed with RIPA Lysis and Extraction Buffer (no. 89900; Thermo Fisher Scientific) containing complete Mini Protease Inhibitor Cocktail (Roche) and, subsequently, centrifuged to collect pellet cell/tissue debris. The lysates were separated by standard SDS-PAGE and analyzed by immunoblotting. Antibodies to the following proteins were used: MACROH2A1 (no. 8551; Cell Signaling Technology), MACROH2A1.1 (no. 12455; Cell Signaling Technology), MACROH2A1.2 (no. 4827; Cell Signaling Technology), p-p65 (no. 3033; Cell Signaling Technology), b-actin (no. 2128; Cell Signaling Technology), CD9 (no. AHS0902; Thermo Fisher Scientific), CD63 (no. MEX002-3; MBL), calnexin (no. ab22595; Abcam), haptoglobin (no. ab131236; Abcam), and flotillin (no. 610821; BD Biosciences).

### Immunohistochemistry

Paraffin-fixed lung tissue samples from three COVID-19 autopsy specimens and three surgical specimens from non-COVID-19 controls at Osaka university hospital were used for immunostaining. Immunohistochemical staining of these samples and a review of the pathologist’s findings were performed by Applied Medical Research Laboratory (Osaka, Japan). Antigen retrieval was performed by autoclaving the samples for 15 min at 125 °C in an EDTA buffer solution (pH 9) after deparaffinization, and endogenous peroxidase activity was blocked with 3% bovine serum albumin at room temperature for 1 h [37]. Slides of the samples were incubated with anti-H2AFY antibody (no. abx103005; Abexa) at 4 °C overnight. They were subsequently incubated with horseradish peroxidase-conjugated anti-rabbit secondary antibody (02–6102; Invitrogen) at room temperature for 30 min.

### Histological analysis

Immunohistochemistry samples stained with anti-H2AFY antibody, as mentioned above, were used to evaluate the percentages of MACROH2A1-positive cells. Images were processed and reconstructed using BZ-X Analyzer software (Keyence Corp., Osaka, Japan) according to the manufacturer’s instructions; in three randomly selected fields of view per sample, the ratio of the number of DAB-positive cells to the number of cells in one field of view was calculated, and the average of the three fields of view was used as the percentage of MACROH2A1-positive cells in that sample. All quantitative measurements were performed in comparable areas under the same optical and light conditions.

### Nanoparticle tracking analysis (NTA)

Analysis of the EVs number and the size distribution was performed by using the NanoSight LM10HS with a blue laser system (NanoSight, Amesbury, UK) as described in a previous report [38]. Briefly, nanoparticle tracking analysis (NTA) was performed on isolated EVs and diluted 20-fold with PBS. For further analysis using the NTA software, all the events were recorded in a 60-s video. The Brownian motion of each particle was tracked between frames to calculate its size using the Stokes − Einstein equation.

### Statistical analysis

Statistical analysis was performed using JMP Pro 13 (SAS Institute Inc., Cary, NC, USA). A two-sided *P* value < 0.05 was considered statistically significant. Categorical and continuous variables were subjected to Fisher’s exact test and unpaired Student’s *t* test, respectively. Principal component and linear regression analyses of proteomics were performed by R.

### Study approval

This study was performed in accordance with the Declaration of Helsinki for medical research involving human subjects. This study was approved by the Ethics Committee of Osaka University Hospital and Toneyama Medical Center, and written informed consent was provided by all patients and healthy controls.

## Results

In this retrospective study, 12 patients with COVID-19 and four healthy controls were subjected to the proteomic analysis. Out of the 12 patients with COVID-19, four non-critical, four critical and non-refractory, and four critical and refractory cases were categorized as Groups 1, 2, and 3, respectively. The baseline characteristics of the patients are presented in Table 1. No significant differences in age or sex and in the frequency of complications were observed between the COVID-19 patient groups or between these patients and healthy controls. The duration of steroid administration at the time of sample collection did not significantly differ between the COVID-19 patient-groups, except for comparisons between Group 1 vs Groups 2 and 3. EVs collected from the serum of both healthy controls and patients with COVID-19 were confirmed by transmission electron microscopy (Supplementary Fig. 1a), and western blotting confirmed the expression of CD9, CD63, and flotillin-1, while calnexin and haptoglobin expressions were negative (Supplementary Fig. 1b). In addition, measurement of the collected EVs by nanoparticle tracking analysis revealed no significant difference in size or number of particles between healthy controls and patients with COVID-19 (Supplementary Fig. 1c, d).

**Table 1:** Baseline characteristics of COVID-19 patients and healthy controls included in the proteomic analysis

	HC	Group 1	Group 2	Group 3	*P* value		
	(*n* = 4)	(*n* = 4)	(*n* = 4)	(*n* = 4)	(Group 3 vs Group 2)	(Groups 2 and 3 vs Group 1)	(COVID-19 vs HC)
Age (year)	73 ± 6.4	74 ± 6.8	64 ± 10.5	81 ± 1.9	0.065	0.83	1
Sex							
Male /female	3 (75)/ 1 (25)	3 (75)/ 1 (25)	3 (75)/ 1 (25)	3 (75)/ 1 (25)	1	1	1
Smoking							
Never/ former/ current	2 (50)/ 0/ 2 (50)	1 (25)/ 3 (75)/ 0	1 (25)/ 3 (75)/ 0	2 (50)/ 2 (50)/ 0	0.47	0.67	0.012
WBC count (10^3^/μL)	6557.5 ± 1686.8	12,660 ± 2429.4	11,320.0 ± 2631.1	7982.5 ± 1050.4	0.087	0.15	0.033
CRP (mg/dL)	0.1 ± 0.1	0.7 ± 0.8	4.2 ± 3.5	3.0 ± 1.9	0.61	0.16	0.013
D-dimer (μg/mL)	NA	3.4 ± 1.7	2.2 ± 0.7	2.1 ± 0.7	0.91	0.6	‒
LDH (U/mL)	192.7 ± 34.7	261.3 ± 9.0	325.0 ± 36.8	286.8 ± 47.2	0.31	0.17	0.0056
Hypertension	2 (50)	0	1 (25)	2 (50)	0.47	0.16	0.35
Diabetes mellitus	0	1 (25)	1 (25)	1 (25)	1	1	0.27
Dyslipidemia	1 (25)	1 (25)	1 (25)	2 (50)	0.47	0.67	0.76
Coronary heart disease	0	0	0	1 (25)	0.29	0.46	0.55
Chronic kidney disease	0	0	0	0	‒	‒	‒
Days after onset	‒	16.0 ± 3.7	14.0 ± 2.9	14.3 ± 1.5	0.9	0.35	‒
Days after dexamethasone	‒	6.8 ± 1.3	9.5 ± 0.9	9.8 ± 1.8	0.83	0.011	‒

*HC* healthy control, *Group 1* non-critical COVID-19, *Group 2* critical-non-refractory COVID-19, *Group 3* critical-refractory COVID-19

Continuous variables are presented as mean ± SD and categorical variables are presented as *n* (%)

**Fig. 1:**
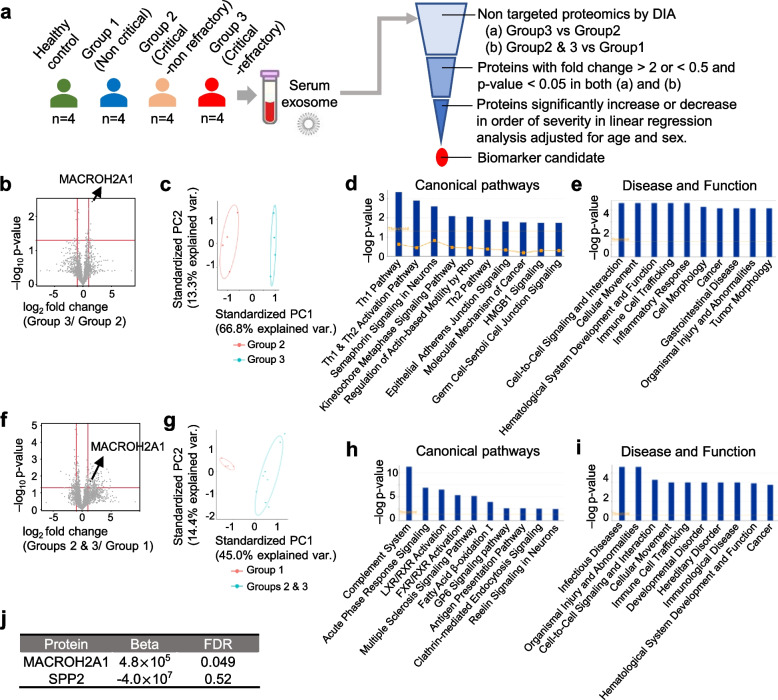
Comparisons of the proteomic profiles of serum EVs by COVID-19 severity and identification of MACROH2A1. **a** Analytical procedures to identify COVID-19 refractory biomarkers. **b** A volcano plot of all identified 2376 serum EV proteins with ≥ 2 total unique peptide count by a non-targeted proteomic analysis comparing Group 2 and 3 cases of COVID-19. **c** Principal component analysis on 12 biomarker candidate proteins distinguishing Group 3 from Group 2. A scatter plot indicates Group 2–Group 3 classes based on the first 2 principal components of the 12 proteins. **d** Top 10 canonical pathways after Ingenuity Pathway Analysis (IPA) for EV proteins significantly upregulated with > 1.5-fold changes or downregulated with < 0.67-fold changes in comparison of Group 2 and 3 cases of COVID-19. **e** Top 10 disease and function after IPA for the EV proteins same as **d**. **f** A volcano plot of all identified 2376 serum EV proteins with ≥ 2 total unique peptide count by a non-targeted proteomic analysis comparing Groups 2 and 3 with Group 1 cases of COVID-19. **g** Principal component analysis performed on 174 biomarker candidate proteins distinguishing Groups 2 and 3 from Group 1. A scatter plot indicates Groups 2 and 3–Group 1 classes based on the first 2 principal components of the 174 proteins. **h** Top 10 canonical pathways after IPA for the EV proteins which were significantly upregulated with > twofold changes or downregulated with < 0.5-fold changes in comparison of Group 2 and 3 cases and Group 1 cases. **i** Top 10 disease and function after IPA for the EV proteins same as **h**. **j** Linear regression analysis adjusted for age and sex to examine whether the proteins with fold change > 2 or < 0.5 and *p* < 0.05 in both **b** and **f** significantly increased or decreased in the order of healthy controls, Group 1, and Groups 2 and 3. β: regression coefficient, FDR: false discovery rate

Non-targeted proteomics by DIA detected a total of 3046 proteins, of which 2376 were identified as proteins with two or more identified peptide fragments. Candidate biomarker molecules were narrowed down from these proteins, as illustrated in Fig. 1a. First, a comparison between critical cases (Group 2 vs Group 3) revealed that 12 proteins were significantly upregulated or downregulated with fold change of < 0.5 or > 2, including Core histone macro-H2A.1 (MACROH2A1) with the lowest *P* value (Fig. 1b, Supplementary Table 1). Of these, six were elevated in Group 3 than in Group 2 cases, while the remaining six were decreased. Principle component analysis (PCA) demonstrated that these identified proteins were well-separated in both groups (Fig. 1c). Additionally, we used the Ingenuity Pathway Analysis (IPA) to explore upstream regulators and perform enrichment analysis, and to increase identification of dysregulated pathways, we loosened the criteria of fold change. IPA on 29 proteins, which were significantly upregulated or downregulated with fold change of < 0.67 or > 1.5 (Supplementary Table 2), revealed enrichment of “Th1 pathway” in an analysis of “canonical pathway” (Fig. 1d), and also revealed enrichment of such pathways as “cell-to-cell signaling and interaction,” “cellular movement,” “hematological system development and function,“ and “immune cell trafficking” in an analysis of “disease and function,” consistent with the pathogenesis of severe COVID-19 (Fig. 1e). Table 2 shows the functional pathways enriched in the “cell-to-cell signaling and interaction” that appear as the first place of the major disease and function categories shown in Fig. 1e. Annotation of these pathways included those related to the function of macrophages, such as “recruitment of macrophages,” and “adhesion of macrophages” (Table 2). The requirements for a biomarker to accurately identify refractory cases are not only to distinguish them from non-refractory cases but also to be able to clearly distinguish them from non-critical cases and to increase or decrease in accordance with the order of increasing severity of the disease. Thus, we next compared the proteomes of critical and non-critical cases (Groups 2 and 3 vs Group 1), and revealed that 174 proteins were significantly upregulated or downregulated with fold change of < 0.5 or > 2, including clathrin light chain A (CLTA) and cytosolic non-specific dipeptidase (CNDP2) which were still significant after Bonferroni correction (*p* = 2.1 × 10^−5^) (Fig. 1f, Supplementary Table 3). Of these, 116 were elevated in Groups 2 and 3 than in Group 1 cases, while the remaining 58 were decreased. Additionally, PCA demonstrated that these identified proteins in both groups were well-isolated (Fig. 1g). In IPA, “complement system” and “acute phase response signaling” were enriched in an analysis of “canonical pathway” (Fig. 1h). Moreover, “infectious disease” and “organismal injury and abnormalities” were enriched in an analysis of “disease and function” (Fig. 1i), and Table 3 shows the functional pathways enriched in “infectious disease” which appear as the first place of the disease and function categories shown in Fig. 1i. Among them the pathway annotated as “severe COVID-19” was the highest ranked. Thus, from the results of the proteomic analysis, there were a total of two proteins, MACROH2A1 and secreted phosphoprotein 24 (SPP2), that were significantly upregulated or downregulated with fold change of < 0.5 or > 2 in the comparison of both Group 2 vs Group 3, and Groups 2 and 3 vs Group 1. Of these, we identified only one protein, MACROH2A1 that significantly increased (FDR < 0.05) in the order of disease severity, in a linear regression analysis adjusted for age and sex (Fig. 1j, Supplementary Table 4, 5). Although MACROH2A1 is a novel molecule that, to our knowledge, has not been previously reported in association with COVID-19, a causal network analysis using IPA on proteins listed in Supplementary Table 2 identified a regulatory relationship between acyl-CoA synthetase 1 (ACSL1) and MACROH2A1 (Supplementary Fig. 2a). ACSL1 was a molecule included in the category of “infectious diseases” and “organismal injuries and abnormalities” that were enriched at the top in Fig. 1i (Table 3). In addition, a causal network analysis by IPA on the proteins listed in Supplementary Table 3 showed a regulatory relationship between COVID-19 and MACROH2A1 via lysine demethylase 2B (KDM2B) (Supplementary Fig. 2b). Collectively, MACROH2A1 was revealed to be a molecule that increased in the order of healthy controls, non-critical cases, and critical cases, and that was able to distinguish between refractory and non-refractory, critical and non-critical, and we considered this protein as the most probable biomarker for predicting refractory COVID-19.

**Table 2:** Functional pathways and categories included in the top enriched pathway in Fig. 1e

Categories	Diseases or functions annotation	*P* value	Molecules	# Molecules
Cell-to-cell signaling and interaction, cellular movement, hematological system development and function, immune cell trafficking, inflammatory response	Recruitment of macrophages	0.0000252	CD8A, ICAM1, NOTCH1, RHOB	4
Cell-to-cell signaling and interaction	Adhesion of breast cancer cell lines	0.000206	CTNND1, ICAM1, SACM1L	3
Cell-to-cell signaling and interaction, cellular movement, hematological system development and function, immune cell trafficking, inflammatory response	Adhesion of macrophages	0.00091	ICAM1, RHOB	2
Cancer, cell-to-cell signaling and interaction, organismal injury and abnormalities	Adhesion of myelomonocytic cells	0.00124	ICAM1	1
Cancer, cell-to-cell signaling and interaction, organismal injury and abnormalities	Activation of tumor cells	0.0013	CTNND1, NOTCH1	2
Cell-to-cell signaling and interaction	Adhesion of connective tissue cells	0.00191	ATP6AP1, ICAM1, RHOB	3
Cancer, cell-to-cell signaling and interaction, organismal injury and abnormalities	Activation of mammary tumor cells	0.00248	CTNND1	1
Cell-mediated immune response, cell-to-cell signaling and interaction, cellular movement, hematological system development and function, immune cell trafficking	Adhesion of regulatory T lymphocytes	0.00248	ICAM1	1
Cell-to-cell signaling and interaction, cellular assembly and organization, hematological system development and function, hypersensitivity response, immune cell trafficking	Cell–cell adhesion of eosinophils	0.00248	ICAM1	1
Cancer, cell-to-cell signaling and interaction, organismal injury and abnormalities	Activation of leukemic blasts	0.00248	NOTCH1	1

**Table 3:** Functional pathways and categories included in the top enriched pathway in Fig. 1i

Categories	Diseases or functions annotation	*P* value	Molecules	# Molecules
Infectious diseases, organismal injury and abnormalities	Severe COVID-19	9.63 × 10^−15^	ACSL1, APOL1, C4A/C4B, C4BPA, C5, C6, FCN2, HLA-DQB1, ITIH3, ITIH4, PF4, PLBD1, SAA1, SAA2, SELL, SPARC, TNFAIP2, TXNIP	18
Infectious diseases, organismal injury and abnormalities	Viral Infection	1.22 × 10^−10^	ABCB1, ABCE1, ACSL1, APOL1, APP, ARPC1b, ARPC5, BGN, C2, C4A/C4B, C4BPA, C5, C6, CAMP, CD14, CD247, CD93, CHMP6, CLTA, COLEC10, DBN1, DDOST, EPS15, FCN2, GPX1, HLA-DQB1, IGHM, ITIH3, ITIH4, ITLN1, JCHAIN, LPL, MUC5B, NPC1L1, PACSIN3, PCSK9, PF4, PLBD1, PLCG2, PNN, PURA, RABEP1, SAA1, SAA2, SELL, SERPINA10, SMPDL3B, SPARC, SPP1, TMX1, TNFAIP2, TPM3, TXNIP, WWP1, ZMPSTE24	55
Infectious diseases, organismal injury and abnormalities	COVID-19	1.44 × 10^−10^	ABCB1, ACSL1, APOL1, C4A/C4B, C4BPA, C5, C6, DBN1, FCN2, GPX1, HLA-DQB1, ITIH3, ITIH4, PCSK9, PF4, PLBD1, SAA1, SAA2, SELL, SPARC, TMX1, TNFAIP2, TXNIP	23
Infectious diseases, organismal injury and abnormalities	Infection by RNA virus	6.59 × 10^−10^	ABCB1, ACSL1, APOL1, APP, ARPC1B, ARPC5, C2, C4A/C4B, C4BPA, C5, C6, CD14, CD93, CLTA, DBN1, DDOST, EPS15, FCN2, GPX1, HLA-DQB1, IGHM, ITIH3, ITIH4, LPL, MUC5B, NPC1L1, PACSIN3, PCSK9, PF4, PLBD1, PURA, SAA1, SAA2, SELL, SPARC, SPP1, TMX1, TNFAIP2, TXNIP, ZMPSTE24	40
Infectious diseases, organismal injury and abnormalities	Infection of mammalian	6.64 × 10^−8^	ADAMTS13, C4A/C4B, CAMP, CD14, CD5L, DCN, HLA-DQB1, IGHM, JCHAIN, MPO, MUC5AC, MUC5B, NRROS, OLFM4, PIGR, PLCG2, SPP1	17
Infectious diseases, inflammatory disease, organismal injury and abnormalities, respiratory disease	Severe acute respiratory syndrome	0.0000417	ACSL1, CAMP, CD14, CD247, GPX1, PNN, SMPDL3B, SPARC, TPM3	9
Infectious diseases, organismal injury and abnormalities	Sepsis	0.0000826	C4A/C4B, C5, CAMP, CD14, FSTL1, HLA-DQB1, LRP1, MPO, MUC5B, SPP1, TGM2	11
Infectious diseases, organismal injury and abnormalities, respiratory disease	Infection of respiratory tract	0.000181	ACSL1, CAMP, CD14, CD247, GPX1, PNN, SAA1, SMPDL3B, SPARC, TPM3	10
Infectious diseases, organismal injury and abnormalities	Tuberculosis	0.000228	ABCB1, CAMP, CD14, CTSG, MPO, OLFM4, SELL, SPP1	8
Infectious diseases, organismal injury and abnormalities	Infection by Neisseria meningitidis	0.000542	C5, C9	2

**Fig. 2:**
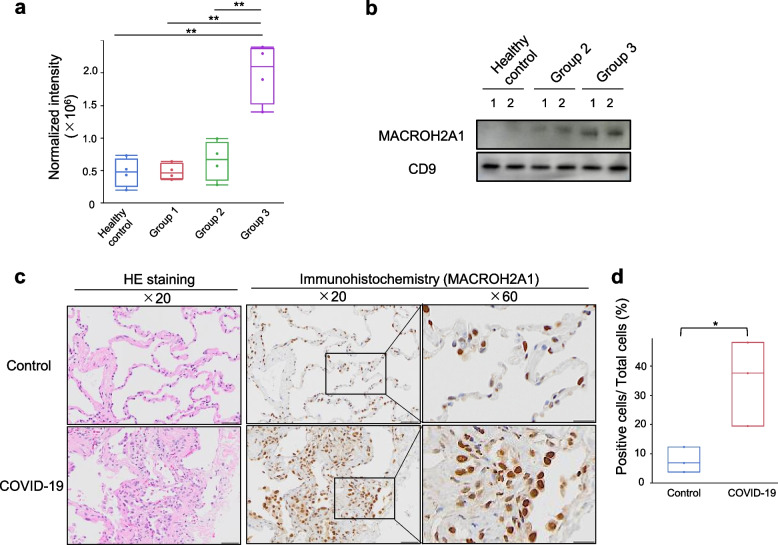
Expression of MACROH2A1 is enhanced in serum EVs and lungs of patients with severe COVID-19. **a** Levels of MACROH2A1 as determined by non-targeted proteomic analysis in patients with COVID-19 and healthy controls. Data are presented as a box plot. **p < 0.001. **b** The levels of MACROH2A1 and CD9, determined by immunoblotting of EVs isolated from sera collected in equal volumes from critical cases with COVID-19 (Groups 2 and 3) and healthy controls. The images were cropped from the original full-length blot image in Supplementary Fig. 10a and b. The data is representative of 2 independent experiments. **c** Histological analysis of lung sections of patients with COVID-19 and controls. Lung sections subjected to hematoxylin–eosin staining are in the left panel and those subjected to immunohistochemistry revealing MACROH2A1-positive cells (brown) are in the right panel. The figures are representative of 3 patients with COVID-19 and 3 controls. **d** Percentages of MACROH2A1-positive cells in lung sections as in **c**, determined using BZ-X Analyzer software. Data are presented as a box plot. *n* = 3 per group (COVID-19), *n* = 3 per group (COVID-19). *p < 0.05

Notably, the quantitative protein value of MACROH2A1 in proteomics was higher in Group 3 cases than in the other groups (Fig. 2a), and this was confirmed in immunoblotting of serum EVs (Fig. 2b). In the ROC analysis for diagnostic performance of MACROH2A1, AUC was 0.63 (0.17–0.93) in the comparison between Group 3 vs Group 2, which exceeded serum CRP and d-dimer (Supplementary Fig. 3a), while in the comparison between Groups 2 and 3 vs Group 1, AUC was 0.84 (0.47–0.97), which was lower than serum CRP though higher than d-dimer (Supplementary Fig. 3b). By immunohistochemical analysis of autopsy lung tissue sections from patients with fatal COVID-19, increased expression of MACROH2A was observed in nuclei of remnant alveolar macrophages as well as type 2 alveolar epithelial cells in fibrosis foci (Fig. 2c, d). Therefore, MACROH2A1 identified by proteomic analysis was elevated in serum EV and locally in the lungs in fatal cases; it can be considered a biomarker candidate that is closely related to the pathogenesis of refractory COVID-19.

**Fig. 3:**
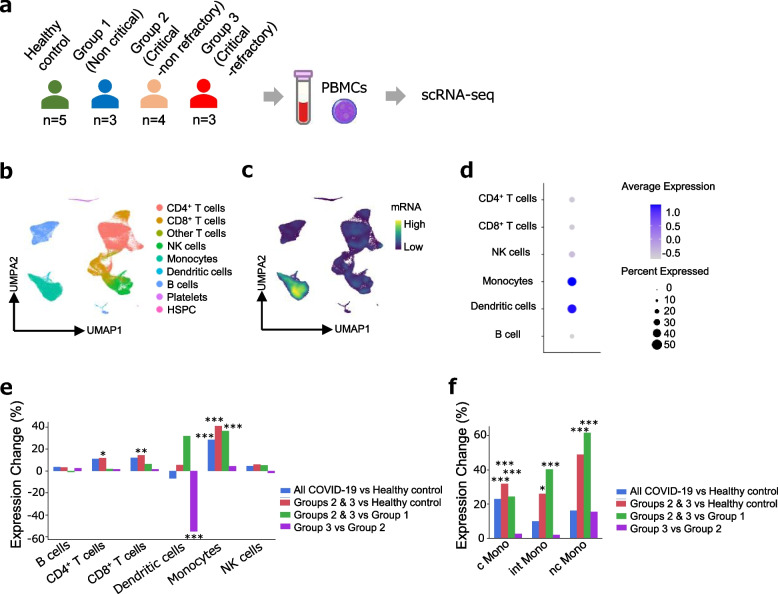
Enhanced expression of *MACROH2A1* in monocytes of severely ill COVID-19 patients revealed by scRNA-seq of PBMCs. **a** Overview of single-cell analysis of PBMCs transcriptome obtained from patients with COVID-19 and healthy controls. **b** UMAP visualization of all 91,830 PBMCs from patients with COVID-19 and healthy controls. **c** Projection of *MACROH2A1* gene expression. **d** Dot plots of *MACROH2A1* expression for each cell of PBMCs. The color is scaled by average expression and the size of the dot is proportional to the percentage of the population expressing *MACROH2A1*. **e** Differential expression analysis of *MACROH2A1* between indicated groups in each cell of PBMCs. **f** Differential expression analysis of *MACROH2A1* between indicated groups in each subpopulation of monocytes. **e,f** The *y*-axis indicates expression changes of overall COVID-19 patient groups relative to healthy controls (blue), Groups 2 and 3 relative to healthy controls (red), Groups 2 and 3 relative to Group 1 (green), and Group 3 and Group 2 (purple), respectively. ncMono: non-classical monocytes, intMono: intermediate monocytes, cMono: classical monocytes, *p < 0.05, **p < 0.01, ***p < 0.001

Subsequently, to investigate the involvement of MACROH2A1 in the pathogenesis of severe COVID-19, we examined its expression in immune cells by scRNA-seq of PBMC. Samples were collected from three non-critical and seven critical (four non-refractory and three refractory) patients with COVID-19, according to the severity groups same as proteomics (Fig. 3a). In the baseline characteristics of the patients (Table 4), there were no significant differences between the groups in sex, duration of steroid administration at the time of specimen collection, and frequency of complications; however, the healthy controls were younger than the patient groups.

**Table 4:** Baseline characteristics of COVID-19 patients and healthy controls included in the scRNA-seq of PBMC

	HC	Group 1	Group 2	Group 3	*P* value			
	(*n* = 4)	(*n* = 3)	(*n* = 4)	(*n* = 3)	(Group 3 vs Group 2)	(Groups 2 and 3 vs Group 1)	(Groups 2 and 3 vs HC)	(COVID-19 vs HC)
Age (year)	28.5 ± 2.6	71.7 ± 5.8	64 ± 10.5	81 ± 2.2	0.064	0.96	< 0.001	< 0.001
Sex								
Male/ female	2 (50)/2 (50)	3 (100)/ 0	3 (75)/ 1 (25)	2 (66.7)/ 1 (33.3)	0.81	0.3	0.48	0.26
Smoking								
Never/ former/ current	4 (100)/ 0/ 0	1 (33.3)/ 0/ 2 (66.7)	2 (50) / 0/ 2 (50)	1 (33.3)/ 0/ 1 (33.3)	0.46	0.7	0.17	0.12
Hypertension	0	2 (66.7)	1 (25)	2 (66.7)	0.27	0.49	0.12	0.07
Diabetes mellitus	0	0	1 (25)	1 (33.3)	0.81	0.3	0.24	0.33
Chronic kidney disease	0	0	0	0	-	-	-	-
Days after onset	-	7.7 ± 1.7	7.8 ± 2.0	8 ± 0.8	0.87	0.89	-	-
Days after corticosteroids	-	1.3 ± 0.9	4 ± 1.6	2 ± 0.8	0.15	0.15	-	-

*HC* healthy control, *Group 1* non-critical COVID-19, *Group 2* critical-non-refractory COVID-19, *Group 3* critical-refractory COVID-19

Continuous variables are presented as mean ± SD and categorical variables are presented as *n* (%)

After the unified single-cell analysis pipeline (see “Methods”), we obtained 91,830 high-quality cells from PBMCs of all the samples. We manually annotated nine cell subsets based on the RNA expression of known marker genes (Fig. 3b, Supplementary Fig. 5a). There was no difference in the percentage of each cell type between the healthy controls and COVID-19 patient-groups of each severity level, except for CD4^+^T cells between Group 3 and the healthy controls (Supplementary Fig. 4a). The expression level of *MACROH2A1* was specifically enhanced in monocytes and dendritic cells (Fig. 3c, d). Differential expression (DE) analysis revealed that *MACROH2A1* expression was generally upregulated in PBMCs, especially in monocytes (Fig. 3e). Moreover, *MACROH2A1* expression was significantly upregulated in critical cases compared to non-critical cases only in monocytes (Fig. 3e). To determine immune cell type specificity of monocytes, we performed clustering and annotation by extracting 18,079 cells belonging to the monocytes subset (Supplementary Fig. 5b). DE analysis showed that *MACROH2A1* expression was significantly upregulated in critical cases compared to non-critical cases or healthy across all of the three monocyte subsets (Fig. 3f), implying that *MACROH2A1* might be involved in the pathogenesis of severe SARS-CoV-2 infection via monocyte function.

**Fig. 4:**
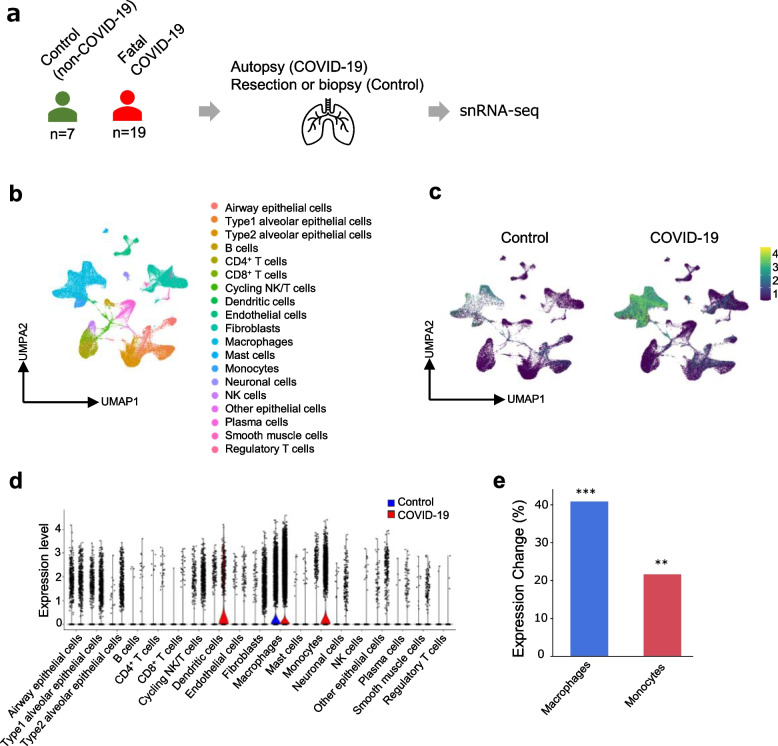
Enhanced expression of *MACROH2A1* in monocytes and macrophages of fatal COVID-19 lungs revealed by snRNA-seq. **a** Overview of single-cell analysis of lung transcriptome obtained from patients with fatal COVID-19 and controls (previous study: Melms et al. (2021)). **b** UMAP embedding of snRNA data analysis of lungs obtained from fatal COVID-19 cases and controls. **c** RNA expression levels (log-normalized) of *MACROH2A1* in each of the controls and fatal COVID-19 cases. **d** Violin plots of *MACROH2A1* expression in each lung cell of controls and fatal COVID-19 cases. **e** Differential expression analysis of *MACROH2A1* in monocytes and macrophages. The *y*-axis indicates expression changes of fatal COVID-19 cases relative to controls. **p < 0.01, ***p < 0.001

We analyzed the previously generated single-nucleus RNA sequencing (snRNA-seq) data of lung tissue using autopsy specimens from 19 patients with COVID-19 and seven control specimens to further examine the involvement of *MACROH2A1* in COVID-19 pneumonia [36]. In our analysis (Fig. 4a), 19 cell types were identified by UMAP visualization (Fig. 4b), and the COVID-19 autopsy specimens revealed a decreased percentage of type 1 and type 2 alveolar epithelial cells and an increased percentage of macrophages, monocytes, and fibroblasts when compared to the control specimens (Supplementary Fig. 4b). Consistent with the findings of PBMC scRNA-seq, *MACROH2A1* was more highly expressed in monocytes and macrophages in the COVID-19 autopsy specimens than in controls (Fig. 4c–e). Collectively, *MACROH2A1* is upregulated in monocytes in circulating immune cells and in the lungs in COVID-19 pneumonia, suggesting that *MACROH2A1* may be involved in the pathogenesis of COVID-19 pneumonia through its function in the monocytes and macrophages.

**Fig. 5:**
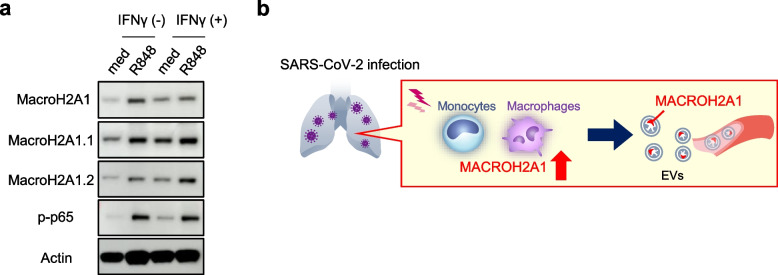
Increased level of MACROH2A1, especially MACROH2A1.2 in response to Toll-like receptor 7 ligand and IFN-gamma. **a** Immunoblot analysis of PMA-differentiated THP-1 cells after treatment with 1 μg /mL R848 with or without 1 μg/mL IFN-gamma for 48 h. med; with no stimulation. The images were cropped from the original full-length blot images in Supplementary Fig. 10c-g. The data are representative of 2 independent experiments. **b** In response to SARS-CoV-2 infection, immune response including TLR signaling and cytokine secretion such as INF-gamma enhances expression of MACROH2A1 in monocytes. Subsequently, MACROH2A1 is secreted in circulating exosomes, which are more abundant in severely ill patients with COVID-19 than in those who are not

Based on these findings, we examined whether stimulation of the innate immune system in response to viral infection, induced MACROH2A1 as in these omics analyses. We stimulated THP-1 cells, a human monocyte cell line, with phorbol myristate acetate/ionomycin (PMA) to induce differentiation into macrophages, and subsequently, added toll-like receptor (TLR) ligands and interferon (IFN)-gamma stimulation. MACROH2A1 was induced by TLR stimulation with R848 (Resiquimod), LPS, and Pam3CSK4 (Fig. 5a, Supplementary Fig. 7b).

Furthermore, MACROH2A1.2, the major isoform of MACROH2A1, was more strongly induced by IFN-gamma stimulation, in addition to R848 stimulation (Fig. 5a). These results were consistent with molecular network analysis performed using KeyMolnet on the EV proteins (Supplementary Fig. 7a). To summarize, MACROH2A1 is induced in monocytic cells in the lungs or circulating blood following COVID-19 infection, possibly in response to viral infection via the TLR signaling pathway or IFN-gamma stimulation (Fig. 5b).

**Fig. 6:**
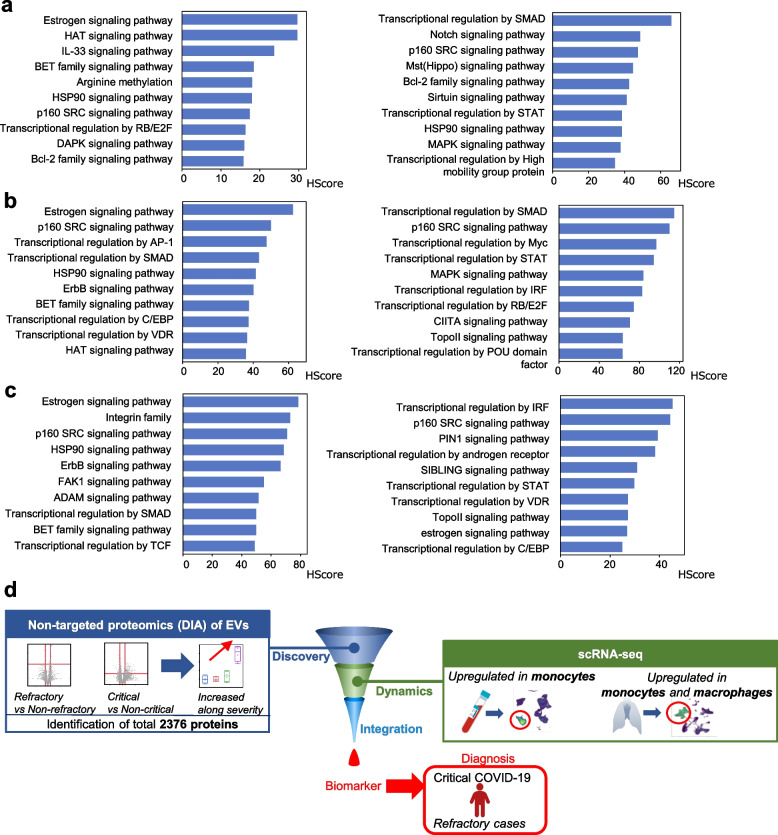
Integration of serum EV proteomics, scRNA-seq of PBMCs, and snRNA-seq of lung tissue. **a–c** KeyMolnet generated a highly complex network of targets with possible relationships by using the “start points and end-points” network search algorithm. In each molecular networks, the top 10 pathways with the highest involvement are listed in order of HScore. Left panel; examination of the molecular network upstream of MACROH2A1. Right panel; examination of the molecular network downstream of MACROH2A1. **a** The top 10 pathways in the networks of EV proteins with *p* < 0.05 and fold change > 1.5 or < 0.67 in comparison of Group 2 and 3 cases. **b** The top 10 pathways in the networks of genes with significantly upregulated or downregulated in differential expression analysis in scRNA-seq of monocytes from PBMCs in comparison of Group 2 and 3 cases and Group 1 cases. **c** The top 10 pathways in the networks of genes with significantly upregulated or downregulated in differential expression analysis in snRNA-seq of monocytes from lungs in comparison of fatal COVID-19 and controls. **d** A graphical abstract of our study. We have identified the protein MACROH2A1, as a potential biomarker for predicting severe COVID-19 infections refractory to anti-inflammatory therapy. First, we have successfully identified several biomarker candidates by performing “next-generation proteomics,” a high-throughput non-targeted quantitative proteomics by data-independent acquisition using serum EVs, and identified MACROH2A1 as the best biomarker molecule among them. Furthermore, scRNA-seq of peripheral blood mononuclear cells and single-nucleus RNA sequencing of lung tissues revealed that this molecule is highly expressed and upregulated in monocytes and macrophages, with pathological pathways which were also reflected in EV proteomics, suggesting its deep involvement in the pathogenesis of severe COVID-19 infections in these cells

Finally, to integrate the overall trends of these serum EVs proteomics, PBMC scRNA-seq, and lung snRNA-seq, molecular network analysis was performed using KeyMolnet on the molecules identified. In the examination of the molecular network upstream of MACROH2A1, its highest involvement in “estrogen signaling” was observed while analyzing the EV proteins with *p* < 0.05 and fold change > 1.5 or < 0.67 in comparison of Group 2 and 3 cases (Fig. 6a, Supplementary Fig. 8a). Moreover, “estrogen signaling” was also highly involved in the analysis of molecules that were significantly upregulated or downregulated in differential expression analysis of monocytes from PBMC scRNA-seq in comparison of Group 2, 3 and Group 1 cases (Fig. 6b, Supplementary Fig. 8b), and in those from lung snRNA-seq in fatal COVID-19 compared to controls (Fig. 6c, Supplementary Fig. 8c). Subsequently, we examined the molecular network downstream of MACROH2A1 and found that “p160 steroid receptor coactivator (SRC) signaling pathway” and “transcriptional regulation by STAT” were commonly involved in the serum EV proteomics (Fig. 6a, Supplementary Fig. 9a), scRNA-seq of PBMCs (Fig. 6b, Supplementary Fig. 9b), and snRNA-seq of lung tissue (Fig. 6c, Supplementary Fig. 9c). The molecules that comprise these upstream and downstream regulatory relationships of MACROH2A1 did include molecules belonging to those pathways (Supplementary Fig. 8a-c, Supplementary Fig. 9a-c). These results revealed a pathway common to all three omics analyses, indicating that MACROH2A1 function in monocytes was reflected to a certain extent in the serum EVs proteomics.

## Discussion

Our high-throughput next-generation proteomic analysis identified MACROH2A1 in EVs as a predictive biomarker of refractory COVID-19 pneumonia. Furthermore, single-cell analysis of PBMCs and lungs indicated that the expression of this molecule, in the monocytes and macrophages, may influence the pathogenesis of severe COVID-19 pneumonia.

Serum EVs are an ideal biomarker source because cargos are stable and associated with certain pathophysiology. Nevertheless, it has several disadvantages such as difficulty of isolation and quantification, especially in case of analyzing small amounts of EV proteins. To overcome these hurdles, we used non-targeted proteomics with high-performance liquid chromatography in this study. Furthermore, the EV isolation using Tim4-affinity method (MagCapture™ Exosome Isolation Kit PS) provides higher EV purity than the gold standard ultracentrifugation method [39]. Hence, in this study, EV isolation based on Tim4-affinity method was combined with non-targeted proteomics by DIA.

To the best of our knowledge, this is the first study to report the association of MACROH2A1 with COVID-19. Although MACROH2A1 has reportedly been detected in serum EVs [40], its detection in serum proteomics is yet to be reported. Our findings may be attributed to the high-throughput proteomics of serum EVs by the DIA method; our proteomics has identified > 2000 proteins with > 2 identified peptide fragments, exceeding the number of proteins identified in previous proteomics reports, wherein only a few hundred to thousand proteins were identified [24, 25]. Conversely, in our previous studies [14, 16, 20], we have identified several thousands of proteins by proteomics of serum EVs. Furthermore, we have demonstrated that they contain a variety of organ-specific EV proteins, including in the lungs [20]. COVID-19 is a respiratory disease; hence, proteomics of serum EVs might serve as a “liquid biopsy” to identify the key molecules of the disease.

Various findings have been presented on the pathogenesis of severe COVID-19 infection. Although mutant strains have emerged over time, an innate immune response to SARS-CoV-2 is induced because the infection has a viral etiology. In innate immune cells, pattern recognition receptors (PRRs) recognize pathogen-associated molecular patterns (PAMPs) and damage-associated molecular patterns (DAMPs) upon SARS-CoV-2 infection [41]. Consequently, type I and type II IFNs are produced, and the production of inflammatory cytokines such as TNF-alpha, IL-1, and IL-18 are induced [42, 43]. Our study identified a potential biomarker molecule, MACROH2A1, which has a fluctuating expression in cells of the monocyte lineage; this forms the basis of innate immune responses, suggesting the involvement of MACROH2A1 in the pathogenesis of COVID-19 that do not change with shifts in epidemic strains due to the emergence of mutant strains, although this hypothesis needs verification, in the future.

MACROH2A1 is a variant of histone H2A, involved in cellular plasticity and proliferation during differentiation and tumorigenesis [44]. It has two isoforms, namely, MACROH2A1.1 and MACROH2A1.2, generated by alternative splicing, and they differ in approximately 30 amino acid residues [45]. Although both MACROH2A1s have many roles in transcriptional regulations, they are known to have many isoform-specific functions. With regard to interaction with ERBB2, a key molecule in estrogen signaling involved in the upstream molecular network of MACROH2A1 (Supplementary Fig. 8a-c); ERBB2 was reported to interact with MACROH2A1.2, but not with MACROH2A1.1 [46], which might result in MACROH2A1.2-specific function in severe COVID-19 pathogenesis. Our findings are consistent with publicly available data on expression levels of MACROH2A1 by cell type [47], wherein it is highly expressed in macrophages and monocytes in the lungs and PBMCs, respectively. Although the involvement of MACROH2A1 in the pathogenesis of severe COVID-19 and its secretion into EVs remains unclear, several possibilities can be considered. A recent study reported that MACROH2A1 binds to the promoter region of *IFNB1* and suppresses typeIIFN production in response to TLR stimulation in monocytes in a zinc finger RNA-binding protein (ZFR)-dependent manner [48]. In our pathway analysis in scRNA-seq of monocytes in PBMCs (Supplementary Fig. 6) and in previous reports [49, 50], type I IFN signaling was suppressed in severe COVID-19 cases, suggesting that MACROH2A1 may be involved in the pathogenesis of refractory COVID-19 through the regulation of typeIIFN production.

Interestingly, we found that the EV proteome reflects a group of molecules and pathways involved in the regulatory relationship of MACROH2A1 in monocytes. These findings are important in addressing whether the novel molecule MACROH2A1 is induced upon COVID-19 infection and further involved in COVID-19 pathogenesis. Notably, estrogen signaling was identified for regulatory relationships upstream of MACROH2A1 by KeyMolnet analysis. A recent study reported a decreased testosterone/estrogen ratio in severe COVID-19 and enhancement of the estrogen signaling pathway in monocytes [51]. These findings indicate that MACROH2A1 induction might be mediated by an enhanced estrogen signaling pathway; however, this requires further investigation. ACSL1 and KDM2B were identified as upstream regulatory molecules of the causal network in IPA. ACSL1, an enzyme that converts free fatty acids to acyl-CoA derivatives, has been reported to be involved in the inflammatory phenotype of monocyte/macrophages [52], consistent with our findings that MACOH2A1 is presumed to function in monocytes. Although there are no COVID-19-related reports, KDM2B is a histone dimethyltransferase that has been reported to bind to the viral epigenome [53] and its expression was affected by viral proteins or conversely regulated viral gene expression [54].

Which pathways in the pathogenesis of COVID-19 are regulated by MACOH2A1? One of the key downstream regulatory relationships of MACOH2A1, the p160/SRC family, is a group of molecules involved in transcriptional regulation [55] and is known to be involved in NF-kB-mediated inflammation and in bacterial infection [56, 57]. It has also been reported that SRCs contribute to HIV reactivation via mTOR and STAT2 [58], and MACROH2A1-SRC family axis may regulate the inflammatory pathogenesis of COVID-19. Regarding the STAT pathway, another important regulatory relationship, it is particularly important that the JAK/STAT (Janus kinase/ Signal Transducer and Activator of Transcription) pathway regulates inflammatory cytokine signaling associated with SARS-CoV-2 infection. In monocytes, STAT1, STAT2 and IFN regulatory factors are activated [59, 60]. The above hypothesized mechanisms provide us with a new perspective on MACROH2A1-mediated severe COVID-19 pathogenesis, and further in vitro and in vivo studies are warranted.

This study has several limitations. First, this is a bi-center study with a small sample size and all patients are Japanese. Therefore, future validation with a large multicenter cohort is required. Second, the biomarker identified in this study is in EVs and cannot be immediately applied in clinical practice. Hence, establishing an ELISA assay system to identify the protein in EVs is necessary.

Although MACROH2A1 is a potential clinical biomarker, its expediency may be further enhanced when used as a multi-biomarker in combination with the other candidate biomarkers such as SPP2, CLTA, and CNDP2 found in this study, allowing for better classification of subtypes, severity, and prognosis, since the value of multiple biomarkers is higher than any single molecule [61]. In addition, integration with other omics, such as genomics and metabolomics, may allow a better understanding and a detailed prediction of the pathogenesis of COVID-19. Although the mechanism of MACROH2A1 regulation of the pathogenesis of severe COVID-19 is unclear, it may function in a cell-specific manner, which makes it a potential therapeutic target with fewer side effects. Furthermore, MACROH2A1 could be used as a companion biomarker to predict the response to such therapy.

## Conclusions

Our findings demonstrate, for the first time, that MACROH2A1 in EVs is a potential biomarker candidate for refractory COVID-19 infection. Furthermore, we propose that it may be involved in the pathogenesis of severe COVID-19 via its function in the monocyte lineage and in the innate immune response to SARS-CoV-2, and additionally, that drug discovery targeting MACROH2A1 could be considered.

## Supplementary Information

**Additional file 1.**

**Additional file 2.**

## Data Availability

All study data are included in the article and additional material.

## Abbreviations

ACSL1: Acyl-CoA synthetase 1
AGC: Automatic gain control
CLTA: Clathrin light chain A
*MACROH2A1*: Core histone macro-H2A.1
COVID-19: Coronavirus disease 2019
CNDP2: Cytosolic non-specific dipeptidase
DAMPs: Damage-associated molecular patterns
DDA: Data-dependent acquisition
DIA: Data-independent acquisition
DE: Differential expression
DEGs: Differentially expressed genes
EV: Extracellular vesicles
FA: Formic acid
GPF: Gas-phase fractionation
HVGs: Highly variable genes
IPA: Ingenuity Pathway Analysis
LC–MS/MS: Liquid chromatography-mass spectrometry
KDM2B: Lysine demethylase 2B
NTA: Nanoparticle tracking analysis
PAMPs: Pathogen-associated molecular patterns
PBMC: Peripheral blood mononuclear cells
PRRs: Pattern recognition receptors
PTS: Phase-transfer surfactant
PMA: Phorbol myristate acetate/ionomycin
PCA: Principal component analysis
SPP2: Secreted phosphoprotein 24
SARS-cov-2: Severe acute respiratory syndrome coronavirus 2
scRNA-seq: Single-cell RNA sequencing
snRNA-seq: Single-nucleus RNA sequencing
TLR: Toll-like receptor
TFA: Trifluoroacetic acid
UMAP: Uniform manifold approximation and projection
UMI: Unique molecular index
