# Supplementary material for: Next-generation proteomics of serum extracellular vesicles combined with single-cell RNA sequencing identifies MACROH2A1 associated with refractory COVID-19
Source: Inflamm Regen. 2022 Nov 30;42:53. doi: 10.1186/s41232-022-00243-5 (PMC9709739; doi:10.1186/s41232-022-00243-5)
Supplement: Supplementary file 1 — Additional file 1. [file 41232_2022_243_MOESM1_ESM.pdf]

Additional file 1 for

Next-generation proteomics of serum extracellular vesicles combined with single-cell RNA sequencing identifies MACROH2A1 associated with refractory COVID-19

Takahiro Kawasaki, Yoshito Takeda*, Ryuya Edahiro, Yuya Shirai, Mari Nogami-Itoh, Takanori Matsuki, Hiroshi Kida, Takatoshi Enomoto, Reina Hara, Yoshimi Noda, Yuichi Adachi, Takayuki Niitsu, Saori Amiya, Yuta Yamaguchi, Teruaki Murakami, Yasuhiro Kato, Takayoshi Morita, Hanako Yoshimura, Makoto Yamamoto, Daisuke Nakatsubo, Kotaro Miyake, Takayuki Shiroyama, Haruhiko Hirata, Jun Adachi, Yukinori Okada, Atsushi Kumanogoh

* Corresponding author: Yoshito Takeda

Email: yoshito@imed3.med.osaka-u.ac.jp

This PDF file includes:

Figures S1 to S12

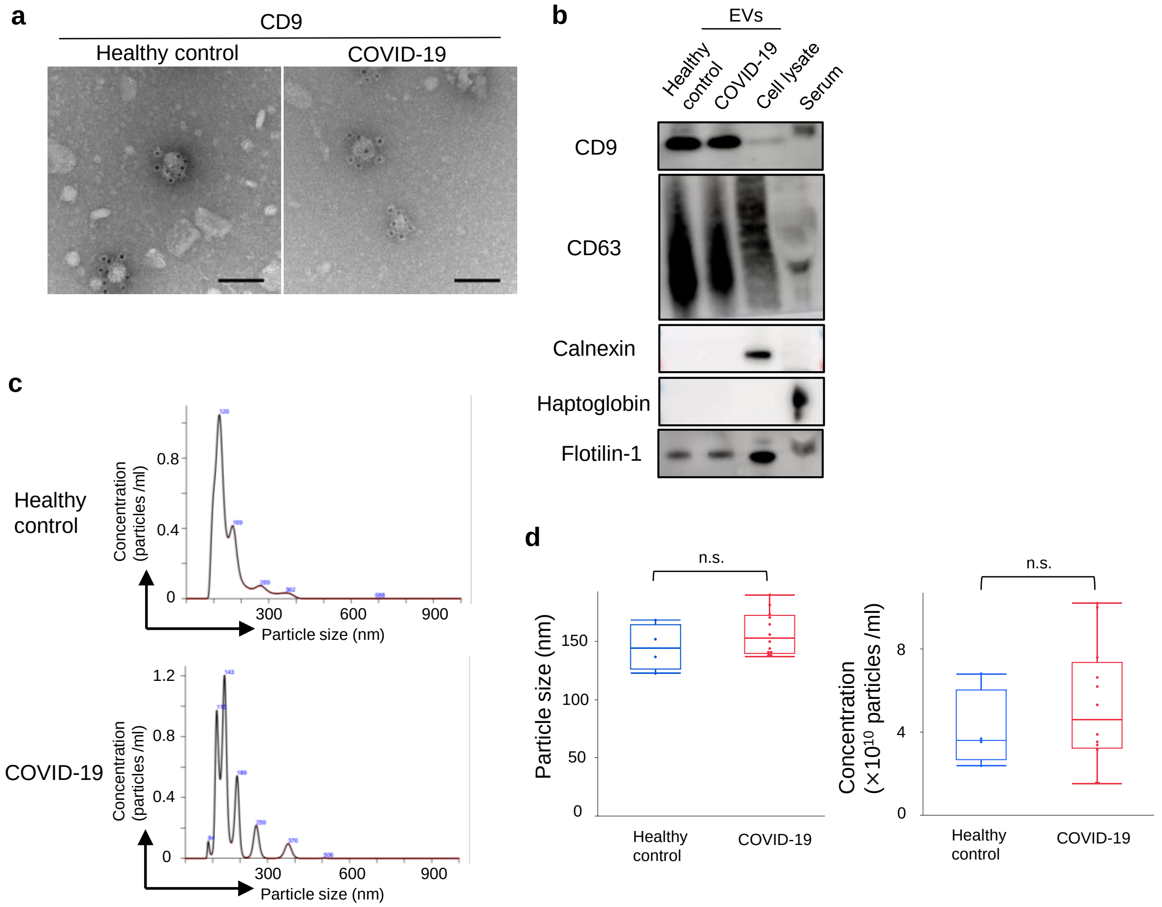

Supplementary Figure 1. Investigation of serum EVs in patients with COVID-19

(a) Transmission electron microscopic images of serum EVs from patients with COVID-19 and healthy controls; immunogold labelling with CD9. Scale bar = 100 nm
(b) Immunoblot analysis of serum EVs, serum from healthy controls and A549 cell lysate. The images were cropped from the original full-length blot images in Supplementary Fig. 11.
(c) Representative figures of distribution curve of serum EVs particle size from patients with COVID-19 and healthy controls, analyzed using NanoSight.
(d) Diameters (left panel) or Concentrations (right panel) of serum EVs from patients with COVID-19 and healthy controls, analyzed using NanoSight. Data are presented as a box plot. n.s.: not significant.

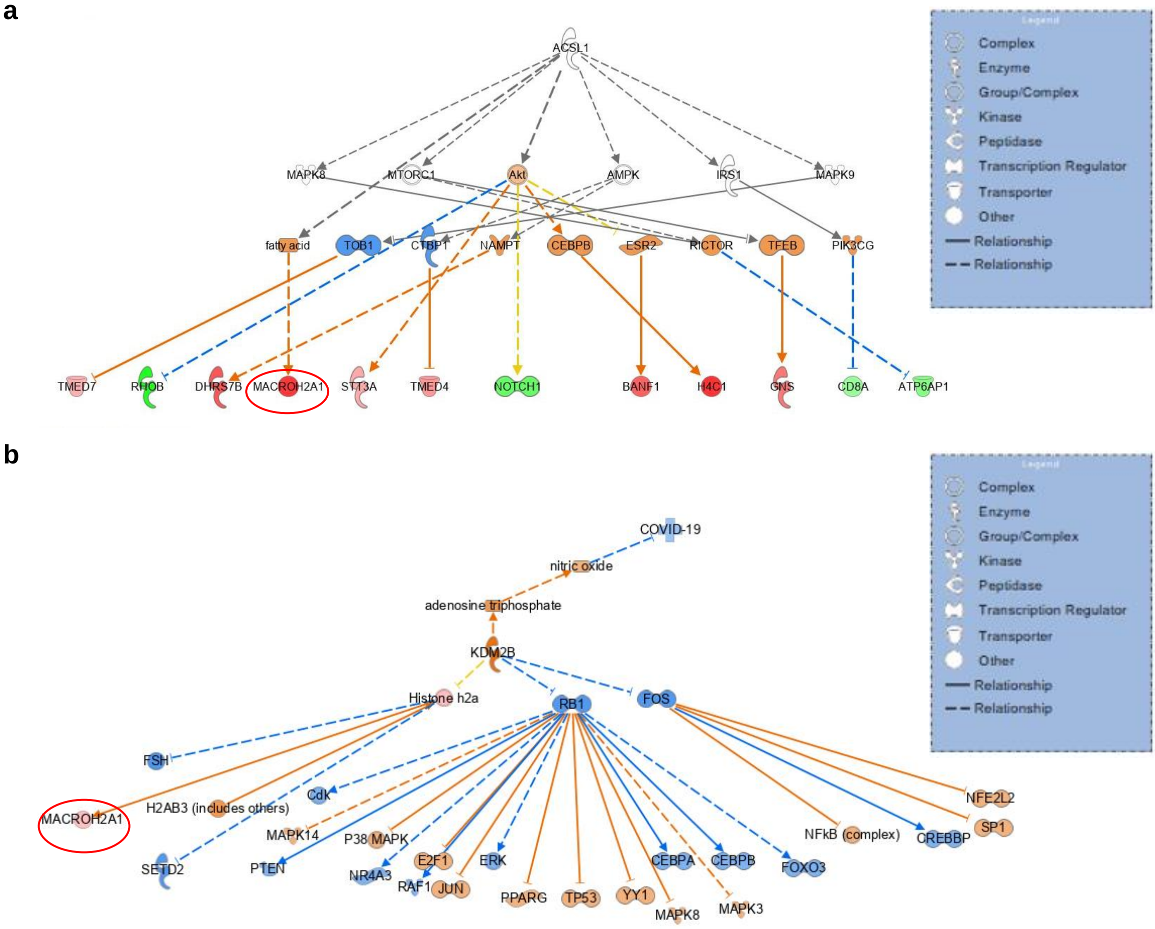

Supplementary Figure 2. Causal regulator molecules and representative causal network of the proteins including MACROH2A1 detected by proteomics

(a) Causal regulator molecules and representative causal network as a result of IPA for the proteins listed in Supplementary Table 2.
(b) Causal regulator molecules and representative causal network as a result of IPA for the proteins listed in Supplementary Table 3.

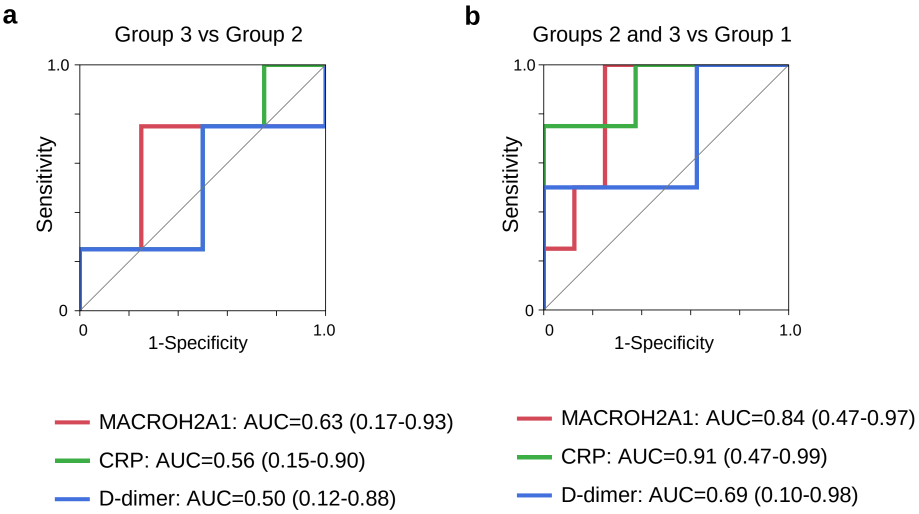

Supplementary Figure 3. ROC analysis for diagnosis of specific COVID-19 severity groups by MACROH2A1

(a, b) AUC values (95% CI) for MACROH2A1 were evaluated by ROC analysis compared to CRP and d-dimer.
(a) ROC analysis for diagnosing Group 3 vs Group 2.
(b) ROC analysis for diagnosing Groups 2 and 3 vs Group 1.

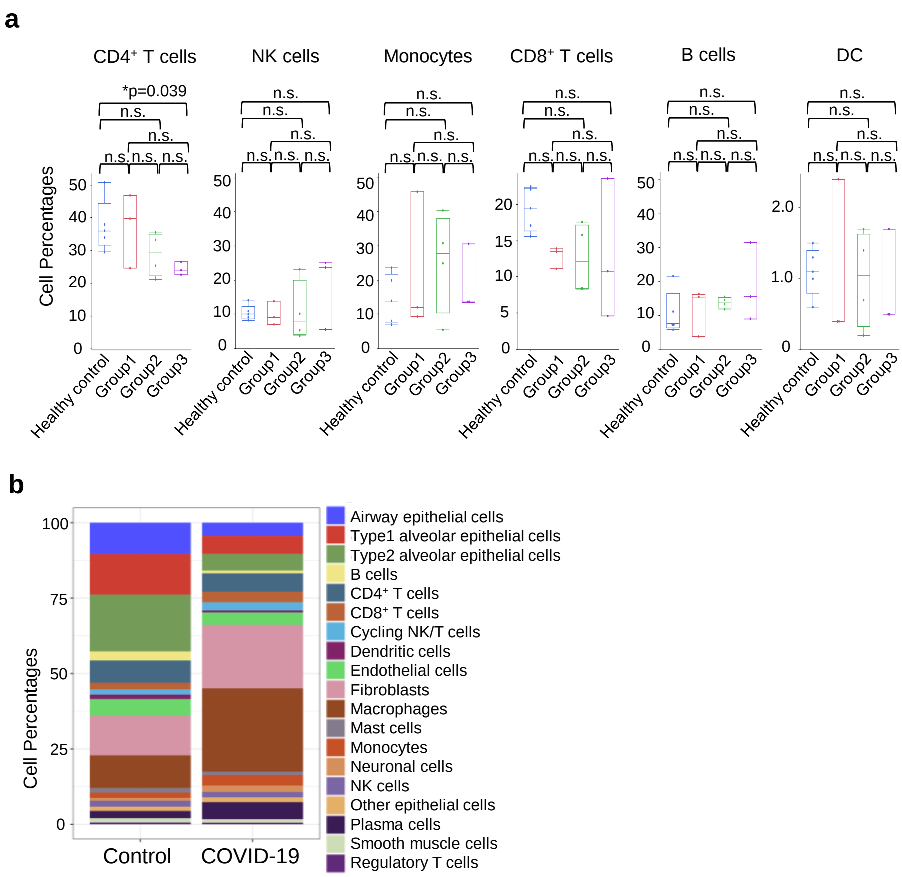

Supplementary Figure 4. Percentage of each cell type in single cell analysis

(a) Boxplots illustrating the percentage of each cell type in Fig. 3b for each stage of disease severity. *p<0.05.
(b) Bar plot illustrating relative contribution of each of the cell types in Fig. 4b for healthy controls and patients with COVID-19.

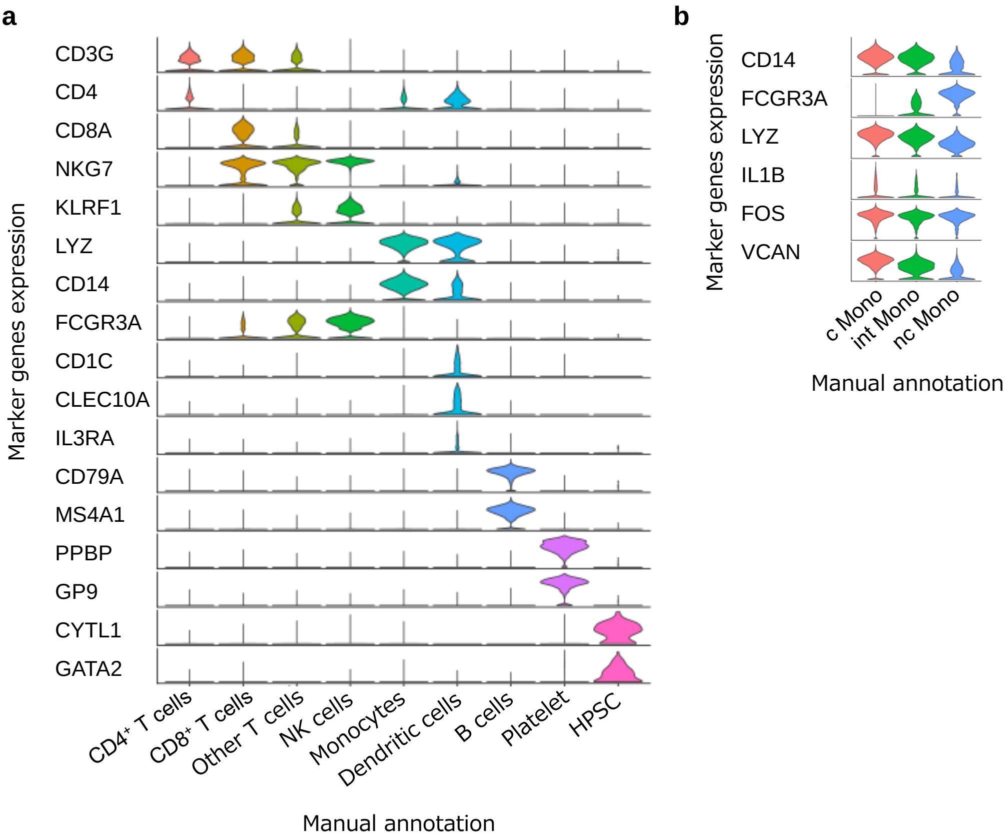

Supplementary Figure 5. Cell type definition in the scRNA-seq of PBMC

(a) Violin plots showing the expression of indicated canonical marker genes in each cell type of PBMC. HSPC: Hematopoietic Stem Progenitor Cell.
(b) Violin plots showing the expression of indicated canonical marker genes in three monocyte subpopulations. ncMono: non-classical monocytes, intMono: intermediate monocytes, cMono: classical monocytes.

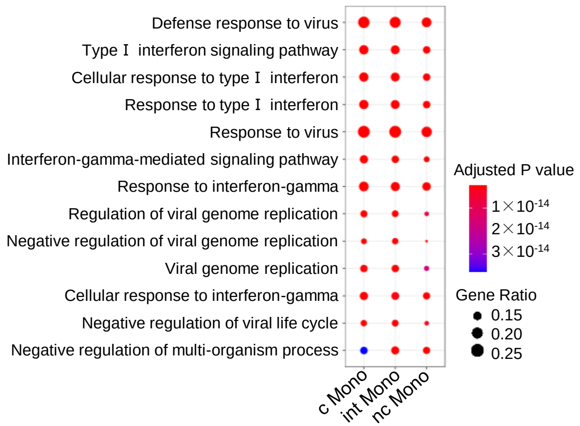

Supplementary Figure 6. Pathway analysis of differential expressed genes in monocytes from COVID-19 patients.

The top 10 enriched biological processes by GO analysis of upregulated differential expressed genes of Group 1 compared to Groups 2 and 3 in the three cell types. Dot color indicates the statistical significance of the enrichment, and dot size represents gene ratio annotated to each term.

a

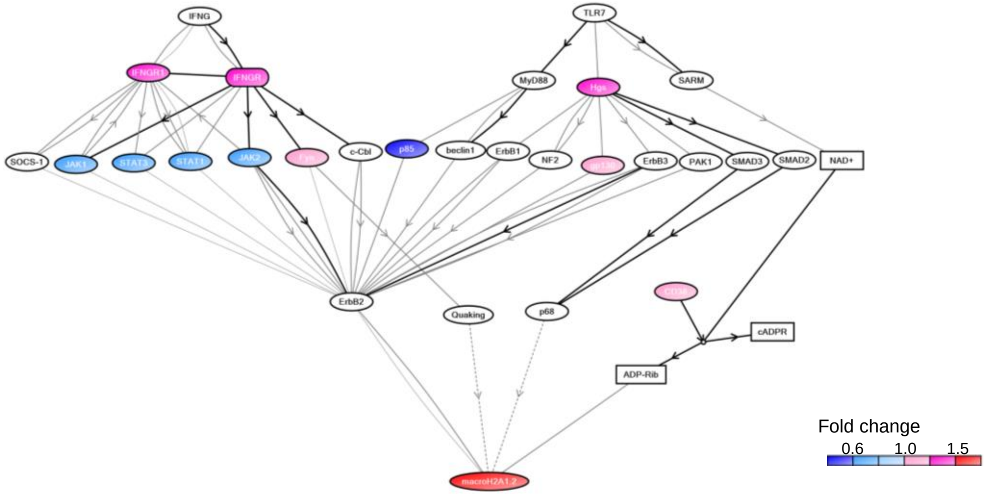

b

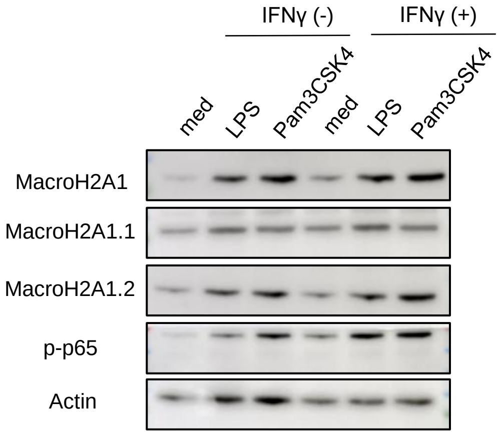

Supplementary Figure 7. Level of MACROH2A1 is increased in response to toll-like receptor ligands and IFN-gamma stimulation.

(a) Molecular network of EV proteins with $p<0.05$ and fold change greater than 1.5 or less than 0.67 focused on the regulatory relationship from TLR7 and IFN-γ to MACROH2A1 in comparison of Group 3 and Group 2. KeyMolnet generated the highly complex network of targets with possible relationships by using the “start points and end-points” network search algorithm. The color indicates fold changes (Group 3/ Group 2).

(b) Immunoblot analysis of PMA-differentiated THP-1 cells after treatment with 100 ng /ml LPS or 1 μg /ml Pam3CSK4 with or without 1 μg /ml IFN-gamma for 48 h. med; with no stimulation. The images were cropped from the original full-length blot images in Supplementary Fig. 12. The data are representative of 2 independent experiments.

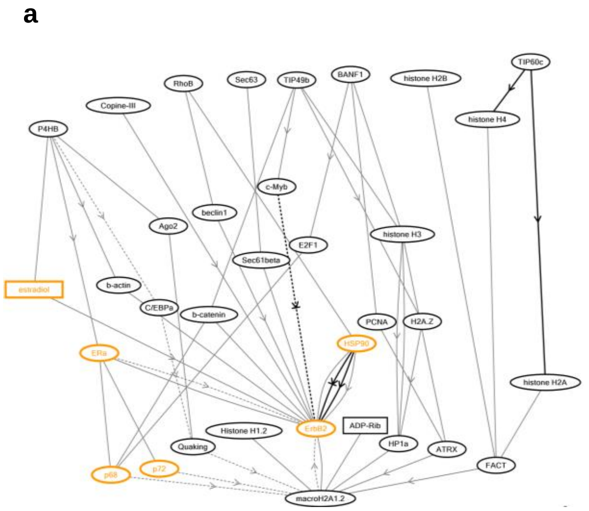

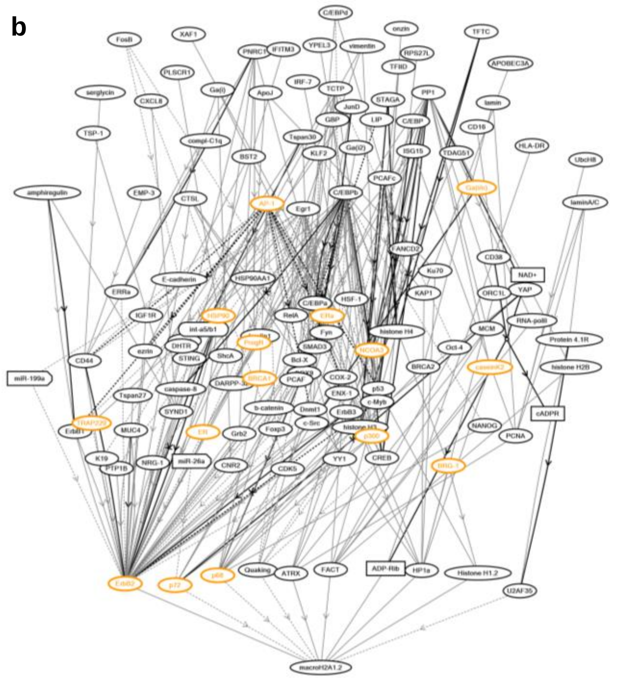

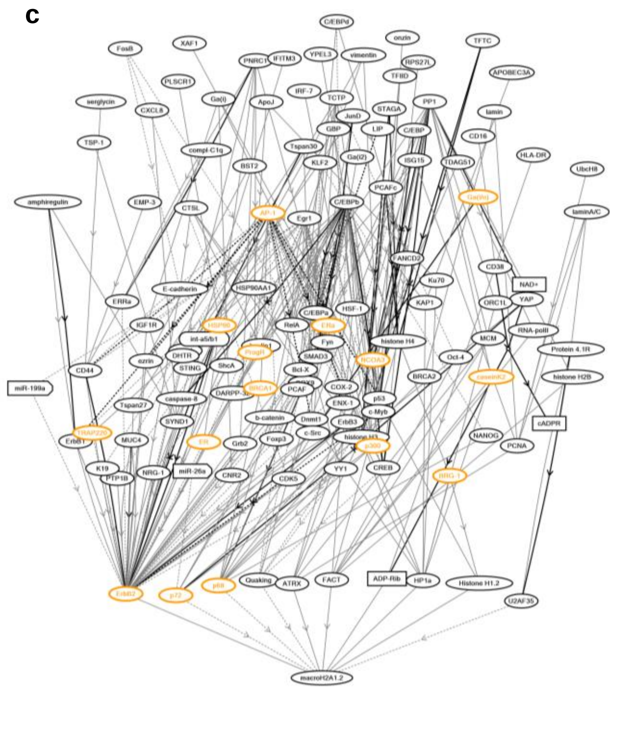

Supplementary Figure 8. Analysis of the upstream molecular network of MACROH2A1 in serum EV proteomics, scRNA-seq of PBMCs, and snRNA-seq of lungs, and the enrichment of "estrogen signaling"-related molecules.

(a-c) KeyMolnet generated a highly complex network of targets with possible relationships by using the "start points and end-points" network search algorithm. Molecular network upstream of MACROH2A1 was illustrated, highlighting molecules involved in "estrogen signaling".

(a) Molecular network of EV proteins with $p<0.05$ and fold change greater than 1.5 or less than 0.67 in comparison of Group 2 and Group 3.

(b) Molecular network of genes with significantly upregulated or downregulated in differential expression analysis in scRNA-seq of monocytes from PBMCs in comparison of Groups 2 and 3 and Group 1.

(c) Molecular network of genes with significantly upregulated or downregulated in differential expression analysis in snRNA-seq of monocytes from lungs in comparison of fatal COVID-19 and controls.

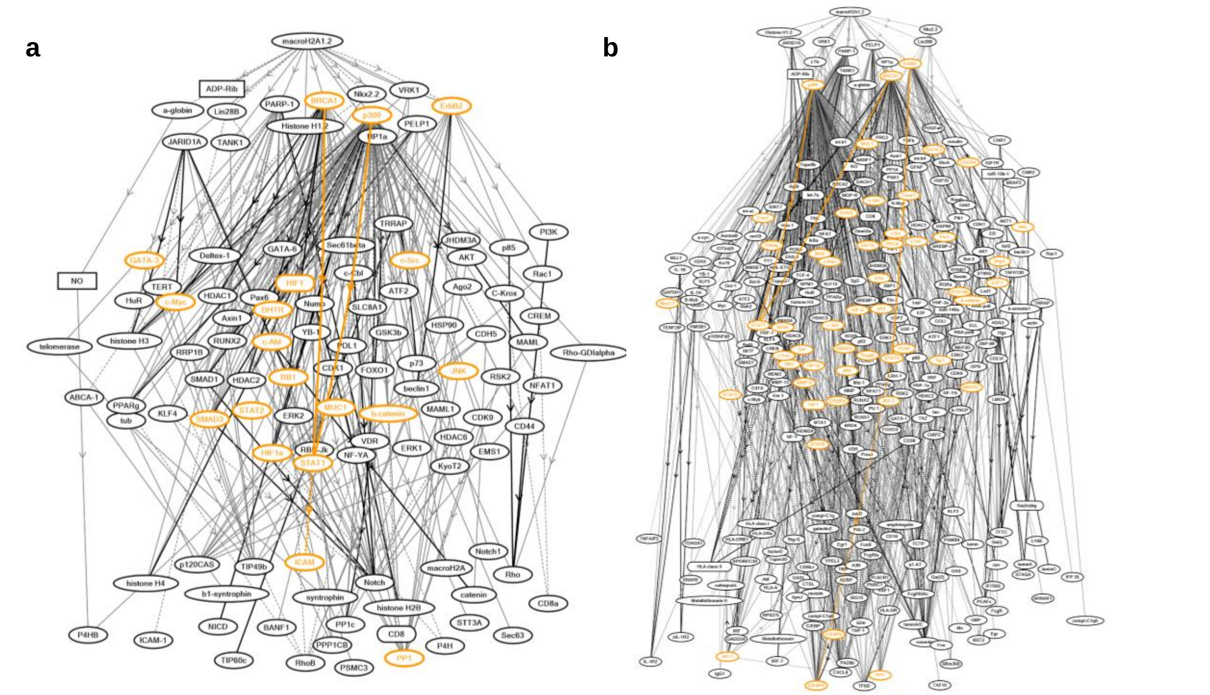

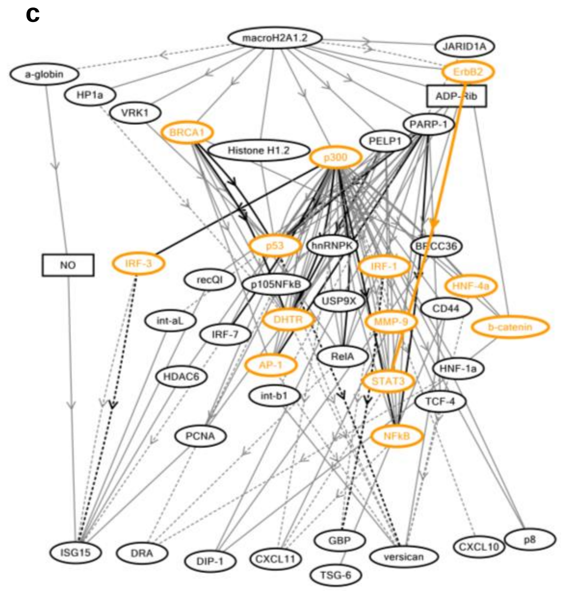

Supplementary Figure 9. Analysis of the downstream molecular network of MACROH2A1 in serum EV proteomics, scRNA-seq of PBMCs, and snRNA-seq of lungs, and the enrichment of "p160 SRC signaling pathway" and "Transcriptional regulation by STAT"-related molecules.

(a-c) KeyMolnet generated a highly complex network of targets with possible relationships by using the "start points and end-points" network search algorithm. Molecular network downstream of MACROH2A1 was illustrated, highlighting molecules involved in "p160 SRC signaling pathway" and "Transcriptional regulation by STAT".

(a) Molecular network of EV proteins with p<0.05 and fold change greater than 1.5 or less than 0.67 in comparison of Group 2 and Group 3.

(b) Molecular network of genes with significantly upregulated or downregulated in differential expression analysis in scRNA-seq of monocytes from PBMCs in comparison of Groups 2 and 3 and Group 1.

(c) Molecular network of genes with significantly upregulated or downregulated in differential expression analysis in snRNA-seq of monocytes from lungs in comparison of fatal COVID-19 and controls.

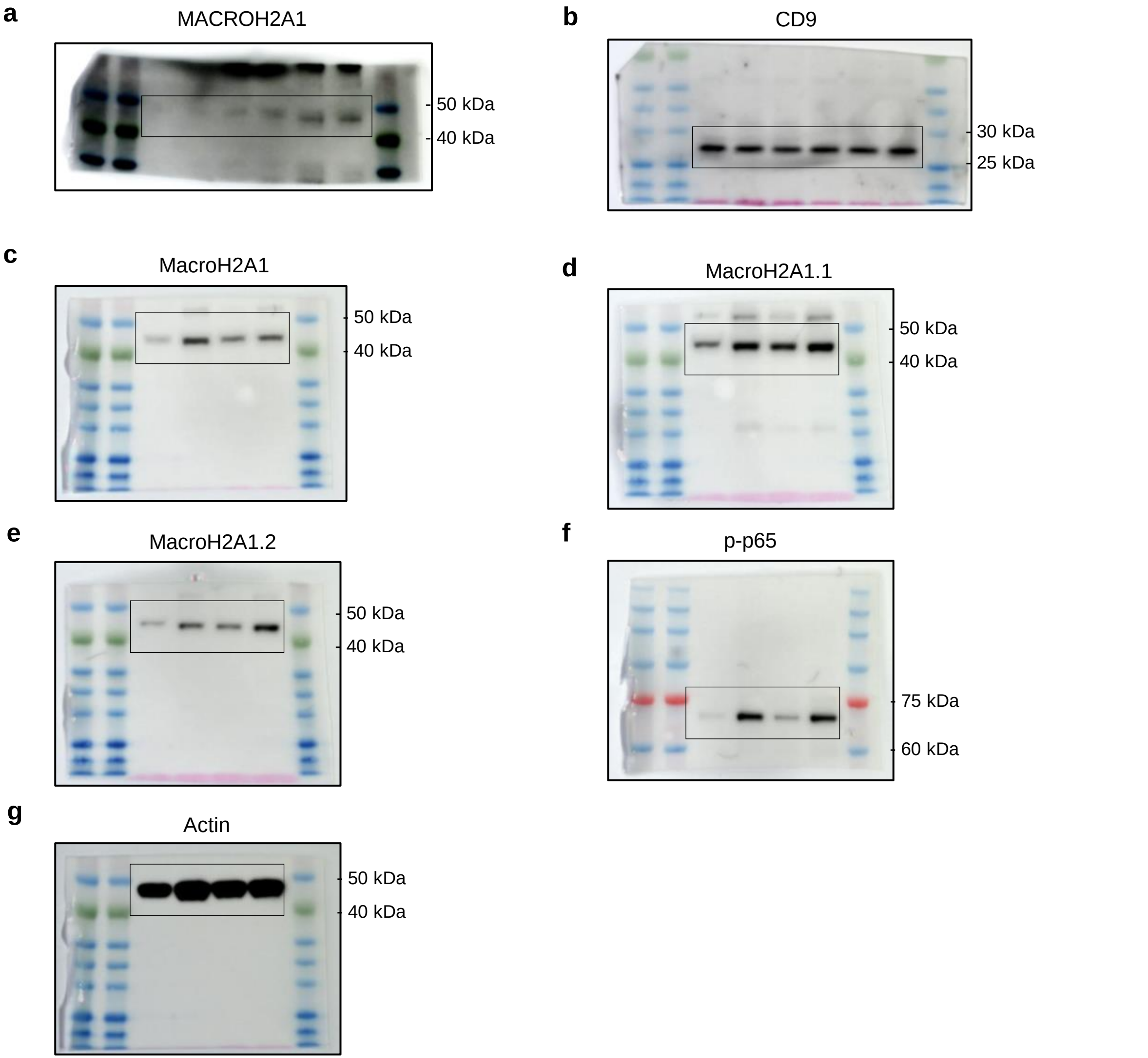

Supplementary Figure 10. The original full-length blot images of Fig. 2b and Fig. 5a.

(a, b) The original full-length blot images of Fig. 2b. The indicated areas in the figures were cropped and shown in Fig. 2b. (c-g) The original full-length blot images of indicated proteins in Fig. 5a. The indicated areas in the figures were cropped and shown in Fig. 5a.

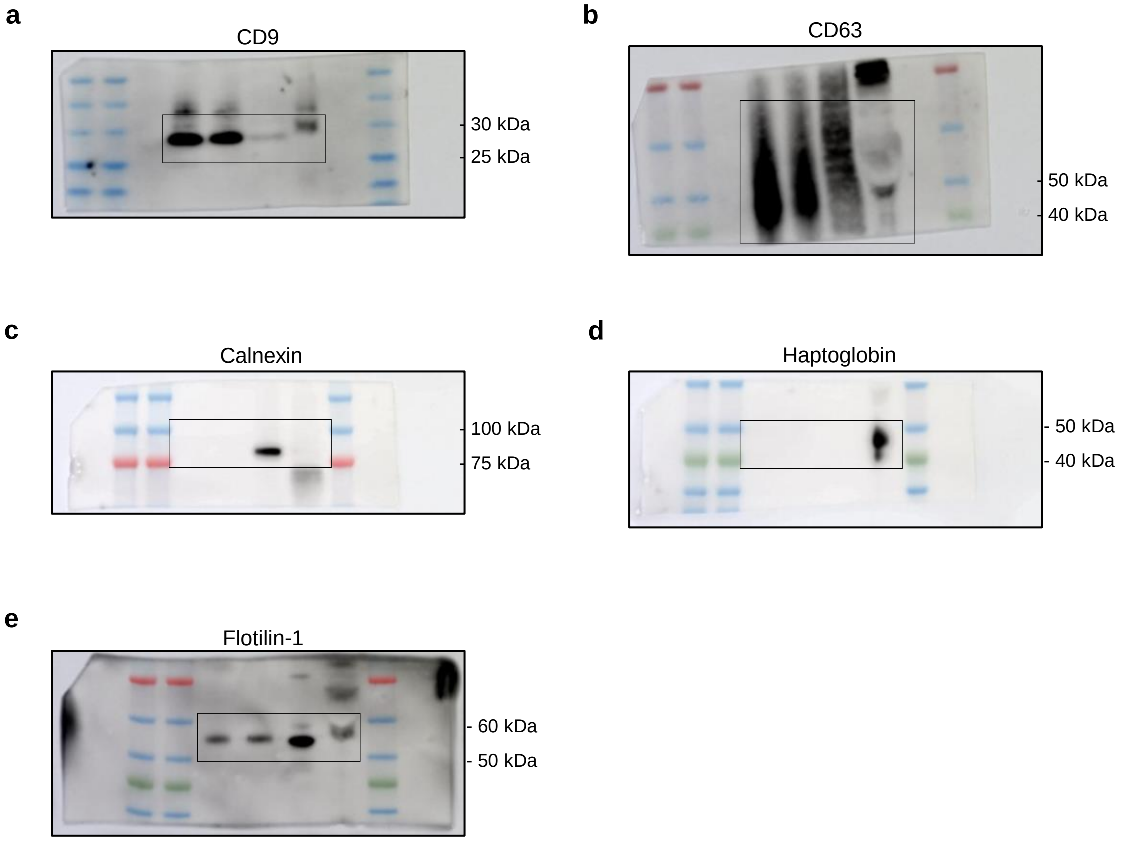

Supplementary Figure 11. The original full-length blot images of Supplementary Fig. 1b.

(a-e) The original full-length blot images of indicated proteins in Supplementary Fig. 1b. The indicated areas in the figures were cropped and were shown in Supplementary Fig. 1b.

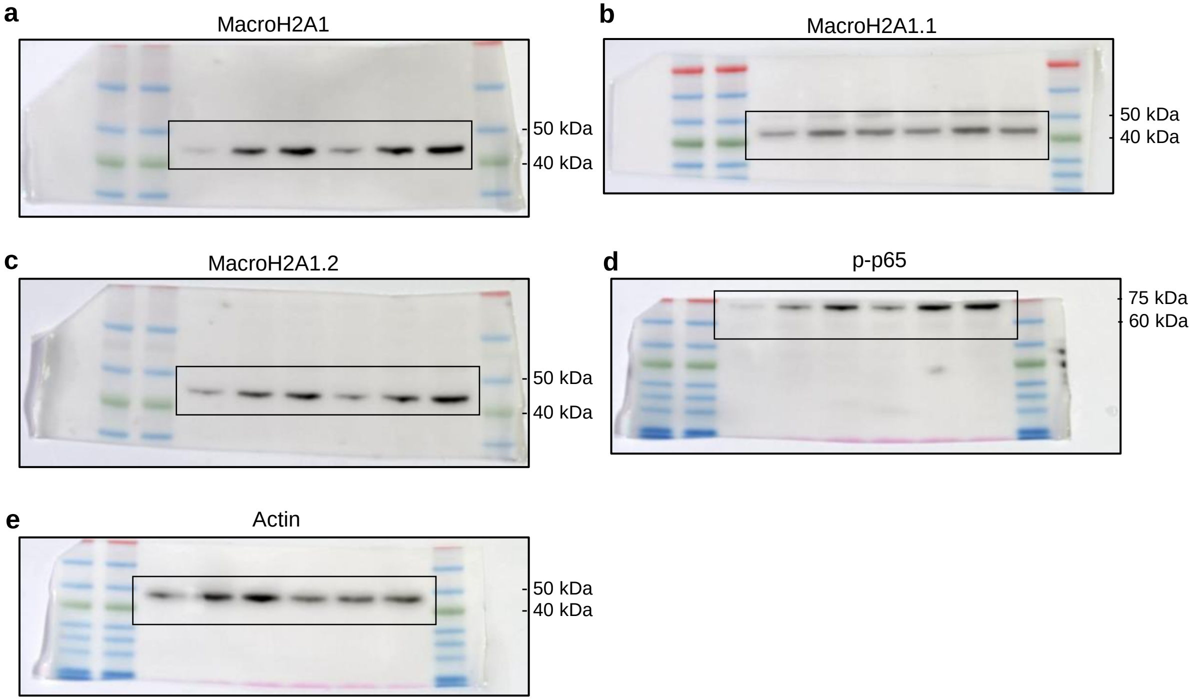

Supplementary Figure 12. The original full-length blot images of Supplementary Fig. 7b.

(a-e) The original full-length blot images of indicated proteins in Supplementary Fig. 7b. The indicated areas in the figures were cropped and were shown in Supplementary Fig. 7b.
