# Supplementary material for: Next-generation proteomics of serum extracellular vesicles combined with single-cell RNA sequencing identifies MACROH2A1 associated with refractory COVID-19
Source: Inflamm Regen. 2022 Nov 30;42:53. doi: 10.1186/s41232-022-00243-5 (PMC9709739; doi:10.1186/s41232-022-00243-5)
Supplement: Supplementary file 2 — Additional file 2. [file 41232_2022_243_MOESM2_ESM.docx]

**Additional file 2 for**

**Next-generation proteomics of serum extracellular vesicles combined with single-cell RNA sequencing identifies MACROH2A1 associated with refractory COVID-19**

Takahiro Kawasaki, Yoshito Takeda*, Ryuya Edahiro, Yuya Shirai, Mari Nogami-Itoh, Takanori Matsuki, Hiroshi Kida, Takatoshi Enomoto, Reina Hara, Yoshimi Noda, Yuichi Adachi, Takayuki Niitsu, Saori Amiya, Yuta Yamaguchi, Teruaki Murakami, Yasuhiro Kato, Takayoshi Morita, Hanako Yoshimura, Makoto Yamamoto, Daisuke Nakatsubo, Kotaro Miyake, Takayuki Shiroyama, Haruhiko Hirata, Jun Adachi, Yukinori Okada, Atsushi Kumanogoh

* Corresponding author: Yoshito Takeda

Email: yoshito@imed3.med.osaka-u.ac.jp

**This Word file includes:**

Tables S1 to S5

**Supplementary Table 1. EV Proteins which were significantly upregulated with more than 2 fold changes or downregulated with less than 0.5 fold changes in comparison of Groups 2 and 3 cases**

| Uniprot ID | Protein name | Total Unique Peptide Count | Fold change | p value |
| --- | --- | --- | --- | --- |
| O75367 | Core histone macro-H2A.1 | 4 | 3.057198 | 0.003716 |
| O75477 | Erlin-1 | 6 | 2.022497 | 0.006137 |
| P17980 | 26S proteasome regulatory subunit 6A | 9 | 0.386072 | 0.007009 |
| Q13884 | Beta-1-syntrophin | 2 | 0.437983 | 0.012452 |
| Q6IAN0 | Dehydrogenase/reductase SDR family member 7B | 2 | 2.58522 | 0.016669 |
| O75531 | Barrier-to-autointegration factor | 2 | 2.422035 | 0.025168 |
| P15586 | N-acetylglucosamine-6-sulfatase | 3 | 2.144367 | 0.043743 |
| Q13103 | Secreted phosphoprotein 24 | 5 | 0.347895 | 0.044007 |
| P06899 | Histone H2B type 1-J | 2 | 2.579805 | 0.04583 |
| P46531 | Neurogenic locus notch homolog protein 1 | 6 | 0.44262 | 0.046227 |
| P62805 | Histone H4 | 9 | 3.175245 | 0.048899 |
| P62745 | Rho-related GTP-binding protein RhoB | 5 | 0.304424 | 0.04937 |

Fold change: mean quantitative values of proteins in EVs of Group 3 cases over those of Group 2 cases.

**Supplementary Table 2. EV Proteins which were significantly upregulated with more than 1.5 fold changes or downregulated with less than 0.67 fold changes in comparison of Groups 2 and 3 cases**

| Uniprot ID | Protein Name | Total Unique Peptide Count | Fold change | p value |
| --- | --- | --- | --- | --- |
| O75367 | Core histone macro-H2A.1 | 4 | 3.05719828 | 0.003715602 |
| O75477 | Erlin-1 | 6 | 2.022497017 | 0.006137085 |
| P01732 | T-cell surface glycoprotein CD8 alpha chain | 6 | 0.584484678 | 0.006432502 |
| P17980 | 26S proteasome regulatory subunit 6A | 9 | 0.386071846 | 0.007008773 |
| O75326 | Semaphorin-7A | 14 | 0.544090686 | 0.007536771 |
| P62140 | Serine/threonine-protein phosphatase PP1-beta catalytic subunit | 3 | 0.608390734 | 0.007843503 |
| O60716 | Catenin delta-1 | 9 | 0.542048893 | 0.011933918 |
| Q13884 | Beta-1-syntrophin | 2 | 0.437982596 | 0.012452268 |
| Q6IAN0 | Dehydrogenase/reductase SDR family member 7B | 2 | 2.585219648 | 0.016668847 |
| O43396 | Thioredoxin-like protein 1 | 3 | 0.519439375 | 0.022069431 |
| Q9NTJ5 | Phosphatidylinositol-3-phosphatase SAC1 | 12 | 1.879940974 | 0.025081145 |
| O75531 | Barrier-to-autointegration factor | 2 | 2.422035328 | 0.02516813 |
| Q6UW02 | Cytochrome P450 20A1 | 3 | 1.923219704 | 0.026808996 |
| O75131 | Copine-3 | 19 | 1.856882883 | 0.02892097 |
| Q7Z7H5 | Transmembrane emp24 domain-containing protein 4 | 4 | 1.789032044 | 0.031278165 |
| P49411 | Elongation factor Tu, mitochondrial | 3 | 1.777372756 | 0.033017176 |
| Q9UGP8 | Translocation protein SEC63 homolog | 7 | 1.916851122 | 0.034081851 |
| P07237 | Protein disulfide-isomerase | 22 | 1.5107859 | 0.040173693 |
| Q15904 | V-type proton ATPase subunit S1 | 2 | 0.528235826 | 0.042345868 |
| P05362 | Intercellular adhesion molecule 1 | 11 | 0.54330709 | 0.042418536 |
| P15586 | N-acetylglucosamine-6-sulfatase | 3 | 2.144367131 | 0.043742529 |
| Q13103 | Secreted phosphoprotein 24 | 5 | 0.347895155 | 0.044006694 |
| P06899 | Histone H2B type 1-J | 2 | 2.579804715 | 0.045829692 |
| P46977 | Dolichyl-diphosphooligosaccharide--protein glycosyltransferase subunit STT3A | 4 | 1.607462882 | 0.045969396 |
| Q9Y3B3 | Transmembrane emp24 domain-containing protein 7 | 6 | 1.548544316 | 0.046047712 |
| P46531 | Neurogenic locus notch homolog protein 1 | 6 | 0.442619719 | 0.046227394 |
| P48444 | Coatomer subunit delta | 3 | 1.815438549 | 0.047300687 |
| P62805 | Histone H4 | 9 | 3.175245458 | 0.048899081 |
| P62745 | Rho-related GTP-binding protein RhoB | 5 | 0.304424152 | 0.049370084 |

Fold change: mean quantitative values of proteins in EVs of Group 3 cases over those of Group 2 cases.

**Supplementary Table 3. EV Proteins which were significantly upregulated with more than 2 fold changes or downregulated with less than 0.5 fold changes in comparison of Groups 2 and 3 and Group 1 cases**

| Uniprot ID | Protein name | Total Unique Peptide Count | Fold change | p value |
| --- | --- | --- | --- | --- |
| P09496 | Clathrin light chain A | 2 | 0.495146922 | 0.000011760 |
| Q96KP4 | Cytosolic non-specific dipeptidase | 11 | 0.446449062 | 0.000017155 |
| Q14761 | Protein tyrosine phosphatase receptor type C-associated protein | 4 | 0.392896084 | 0.000274952 |
| P07203 | Glutathione peroxidase 1 | 7 | 0.414629931 | 0.000707465 |
| P48426 | Phosphatidylinositol 5-phosphate 4-kinase type-2 alpha | 5 | 0.474245863 | 0.00103489 |
| Q6P4A8 | Phospholipase B-like 1 | 7 | 0.408005636 | 0.001615328 |
| P20963 | T-cell surface glycoprotein CD3 zeta chain | 10 | 0.417951768 | 0.001945403 |
| P40197 | Platelet glycoprotein V | 6 | 2.786319829 | 0.001946691 |
| Q93084 | Sarcoplasmic/endoplasmic reticulum calcium ATPase 3 | 18 | 0.466460624 | 0.002234181 |
| P0C0L5 | Complement C4-B | 5 | 3.772157434 | 0.002593971 |
| Q15485 | Ficolin-2 | 4 | 7.631114274 | 0.002728807 |
| O43852 | Calumenin | 2 | 3.314772819 | 0.002841397 |
| Q9ULC5 | Long-chain-fatty-acid--CoA ligase 5 | 4 | 0.48697117 | 0.002939831 |
| Q9UBW5 | Bridging integrator 2 | 4 | 0.373549914 | 0.003564746 |
| P14543 | Nidogen-1 | 18 | 3.27196552 | 0.003683007 |
| P02776 | Platelet factor 4 | 3 | 19.6515799 | 0.003957508 |
| Q9NRF8 | CTP synthase 2 | 2 | 0.474354574 | 0.004073419 |
| Q9UHC9 | NPC1-like intracellular cholesterol transporter 1 | 9 | 5.886042269 | 0.004333465 |
| P49913 | Cathelicidin antimicrobial peptide | 5 | 2.257020914 | 0.004349697 |
| P49006 | MARCKS-related protein | 2 | 0.485780186 | 0.005029038 |
| Q14112 | Nidogen-2 | 6 | 4.922993587 | 0.005197864 |
| Q15067 | Peroxisomal acyl-coenzyme A oxidase 1 | 2 | 0.077477417 | 0.005358944 |
| P0DJI8 | Serum amyloid A-1 protein | 4 | 9.7755116 | 0.005512313 |
| P14151 | L-selectin | 5 | 3.763781636 | 0.00551547 |
| Q16270 | Insulin-like growth factor-binding protein 7 | 3 | 24.0617825 | 0.006047373 |
| Q14624 | Inter-alpha-trypsin inhibitor heavy chain H4 | 37 | 2.82550884 | 0.006545756 |
| P01871 | Immunoglobulin heavy constant mu | 20 | 6.260130918 | 0.006610211 |
| P35052 | Glypican-1 | 6 | 10.08390434 | 0.007004957 |
| Q8WUJ3 | Cell migration-inducing and hyaluronan-binding protein | 3 | 3.766924727 | 0.00701574 |
| Q9Y5C1 | Angiopoietin-related protein 3 | 2 | 7.455295676 | 0.007156221 |
| Q8TAA9 | Vang-like protein 1 | 4 | 5.467322701 | 0.007251353 |
| O00468 | Agrin | 6 | 7.25880007 | 0.007256197 |
| Q9H0M0 | NEDD4-like E3 ubiquitin-protein ligase WWP1 | 5 | 2.595670357 | 0.007473477 |
| O75844 | CAAX prenyl protease 1 homolog | 2 | 0.443103574 | 0.007647786 |
| P01833 | Polymeric immunoglobulin receptor | 17 | 5.396933802 | 0.0077304 |
| Q8WWA0 | Intelectin-1 | 7 | 0.147282385 | 0.007963557 |
| O95865 | N(G),N(G)-dimethylarginine dimethylaminohydrolase 2 | 5 | 0.434605395 | 0.007993406 |
| Q9H8L6 | Multimerin-2 | 3 | 6.949110348 | 0.009113535 |
| Q13103 | Secreted phosphoprotein 24 | 5 | 0.218321615 | 0.009238257 |
| Q00577 | Transcriptional activator protein Pur-alpha | 2 | 0.440894511 | 0.009560463 |
| P10451 | Osteopontin | 2 | 4.096231584 | 0.009788127 |
| Q12841 | Follistatin-related protein 1 | 4 | 4.556024486 | 0.010422595 |
| P05067 | Amyloid-beta precursor protein | 8 | 2.028282835 | 0.010539784 |
| Q8NBP7 | Proprotein convertase subtilisin/kexin type 9 | 9 | 10.57226493 | 0.010873813 |
| Q8IVB4 | Sodium/hydrogen exchanger 9 | 3 | 3.400838711 | 0.011327367 |
| P01920 | HLA class II histocompatibility antigen, DQ beta 1 chain | 4 | 7.277786268 | 0.012243711 |
| O15143 | Actin-related protein 2/3 complex subunit 1B | 11 | 0.489828444 | 0.012492093 |
| Q9UI12 | V-type proton ATPase subunit H | 5 | 0.377577372 | 0.013093421 |
| P06858 | Lipoprotein lipase | 6 | 6.803126521 | 0.013438529 |
| P01591 | Immunoglobulin J chain | 6 | 5.539364516 | 0.013768315 |
| O14791 | Apolipoprotein L1 | 9 | 2.628003678 | 0.014095495 |
| Q9NPY3 | Complement component C1q receptor | 5 | 2.039522668 | 0.014733193 |
| Q9GZY6 | Linker for activation of T-cells family member 2 | 2 | 2.626801445 | 0.014857755 |
| Q8WWZ8 | Oncoprotein-induced transcript 3 protein | 14 | 3.508487849 | 0.015178675 |
| P39060 | Collagen alpha-1(XVIII) chain | 11 | 28.62147138 | 0.015801392 |
| P08311 | Cathepsin G | 5 | 3.264924466 | 0.015905267 |
| Q8NBM8 | Prenylcysteine oxidase-like | 2 | 0.226205979 | 0.016246455 |
| Q13790 | Apolipoprotein F | 4 | 7.038941688 | 0.016640324 |
| Q9H3N1 | Thioredoxin-related transmembrane protein 1 | 7 | 0.347420172 | 0.016799842 |
| P61086 | Ubiquitin-conjugating enzyme E2 K | 3 | 2.027801605 | 0.017014457 |
| Q96CS7 | Pleckstrin homology domain-containing family B member 2 | 4 | 2.208764479 | 0.017038778 |
| Q03591 | Complement factor H-related protein 1 | 3 | 3.725718238 | 0.017188833 |
| Q99969 | Retinoic acid receptor responder protein 2 | 2 | 4.593915097 | 0.017191192 |
| Q8NCG7 | Diacylglycerol lipase-beta | 5 | 0.422071396 | 0.017606994 |
| Q9H307 | Pinin | 2 | 0.331297039 | 0.017627607 |
| Q16706 | Alpha-mannosidase 2 | 7 | 2.438228813 | 0.01790865 |
| P07585 | Decorin | 8 | 14.68232107 | 0.018227688 |
| P35268 | 60S ribosomal protein L22 | 2 | 2.216391834 | 0.018269202 |
| Q16643 | Drebrin | 5 | 2.603672607 | 0.018397525 |
| P98088 | Mucin-5AC | 17 | 2.627848853 | 0.01852947 |
| P10643 | Complement component C7 | 34 | 4.831457938 | 0.018912634 |
| O43866 | CD5 antigen-like | 8 | 6.070874674 | 0.019075027 |
| Q9BZ29 | Dedicator of cytokinesis protein 9 | 6 | 2.368446915 | 0.019471474 |
| Q9H3M7 | Thioredoxin-interacting protein | 6 | 0.464558617 | 0.019547178 |
| Q9Y673 | Dolichyl-phosphate beta-glucosyltransferase | 2 | 0.49020013 | 0.019643677 |
| Q9HC84 | Mucin-5B | 22 | 2.088749273 | 0.019915028 |
| Q5T2W1 | Na(+)/H(+) exchange regulatory cofactor NHE-RF3 | 4 | 3.16875051 | 0.020110437 |
| Q96IU4 | Protein ABHD14B | 2 | 4.328712427 | 0.020320314 |
| P02675 | Fibrinogen beta chain | 15 | 2.060573042 | 0.020326827 |
| Q92496 | Complement factor H-related protein 4 | 3 | 3.708495117 | 0.020412869 |
| O14638 | Ectonucleotide pyrophosphatase/phosphodiesterase family member 3 | 8 | 3.547461592 | 0.020590021 |
| O75367 | Core histone macro-H2A.1 | 4 | 2.742509102 | 0.020678143 |
| Q9UM47 | Neurogenic locus notch homolog protein 3 | 3 | 5.902616895 | 0.020928171 |
| P21810 | Biglycan | 10 | 30.84111477 | 0.021153687 |
| Q7RTS7 | Keratin, type II cytoskeletal 74 | 2 | 2.638423032 | 0.021220937 |
| P61221 | ATP-binding cassette sub-family E member 1 | 4 | 3.450608628 | 0.0213892 |
| P36959 | GMP reductase 1 | 5 | 0.464274328 | 0.021584396 |
| P46108 | Adapter molecule crk | 2 | 7.987457727 | 0.021682816 |
| P61201 | COP9 signalosome complex subunit 2 | 3 | 19.14696017 | 0.021890179 |
| P01031 | Complement C5 | 67 | 4.474141278 | 0.021939441 |
| P0DJI9 | Serum amyloid A-2 protein | 5 | 13.37674547 | 0.021963189 |
| Q9UKK3 | Protein mono-ADP-ribosyltransferase PARP4 | 24 | 11.57858397 | 0.022125643 |
| Q16629 | Serine/arginine-rich splicing factor 7 | 2 | 0.3538745 | 0.022344831 |
| Q9UKS6 | Protein kinase C and casein kinase substrate in neurons protein 3 | 6 | 4.330835691 | 0.02482199 |
| P08571 | Monocyte differentiation antigen CD14 | 8 | 4.51098073 | 0.025037453 |
| O94832 | Unconventional myosin-Id | 4 | 3.638581617 | 0.025377849 |
| Q9Y3F4 | Serine-threonine kinase receptor-associated protein | 4 | 2.718204509 | 0.026456175 |
| Q96FZ7 | Charged multivesicular body protein 6 | 2 | 0.42267429 | 0.026830148 |
| Q92485 | Acid sphingomyelinase-like phosphodiesterase 3b | 2 | 4.21948741 | 0.026959892 |
| O15511 | Actin-related protein 2/3 complex subunit 5 | 4 | 0.482258042 | 0.026965511 |
| Q96RF0 | Sorting nexin-18 | 6 | 11.26740694 | 0.026980166 |
| P22234 | Multifunctional protein ADE2 | 11 | 0.429790908 | 0.0270817 |
| Q96R05 | Retinoid-binding protein 7 | 2 | 2.72760361 | 0.027679413 |
| P02748 | Complement component C9 | 24 | 4.600868866 | 0.028053461 |
| P16885 | 1-phosphatidylinositol 4,5-bisphosphate phosphodiesterase gamma-2 | 6 | 2.609897204 | 0.028121346 |
| P21980 | Protein-glutamine gamma-glutamyltransferase 2 | 7 | 0.291044179 | 0.029622645 |
| P49758 | Regulator of G-protein signaling 6 | 3 | 4.132426928 | 0.029736364 |
| P25789 | Proteasome subunit alpha type-4 | 4 | 0.447237809 | 0.029802083 |
| Q15276 | Rab GTPase-binding effector protein 1 | 11 | 10.87864493 | 0.029905303 |
| O15198 | Mothers against decapentaplegic homolog 9 | 2 | 0.497961439 | 0.03017877 |
| P51659 | Peroxisomal multifunctional enzyme type 2 | 16 | 0.276203898 | 0.030906697 |
| Q9H1E1 | Ribonuclease 7 | 2 | 0.370451912 | 0.031215814 |
| P06681 | Complement C2 | 23 | 4.506107361 | 0.031500548 |
| Q96AC1 | Fermitin family homolog 2 | 10 | 2.612501514 | 0.031857529 |
| Q03169 | Tumor necrosis factor alpha-induced protein 2 | 6 | 5.291816375 | 0.032126573 |
| Q86YC3 | Transforming growth factor beta activator LRRC33 | 2 | 6.203834456 | 0.032459662 |
| Q9BZG1 | Ras-related protein Rab-34 | 2 | 2.012282128 | 0.03253566 |
| P06331 | Immunoglobulin heavy variable 4-34 | 3 | 7.60777064 | 0.032682793 |
| Q9Y639 | Neuroplastin | 4 | 0.443846657 | 0.032898013 |
| Q9Y6Z7 | Collectin-10 | 7 | 3.806631877 | 0.033207906 |
| P07358 | Complement component C8 beta chain | 20 | 4.675673131 | 0.033338392 |
| P13671 | Complement component C6 | 32 | 4.103840198 | 0.033833235 |
| P04003 | C4b-binding protein alpha chain | 27 | 2.332462516 | 0.033851066 |
| Q9UJC5 | SH3 domain-binding glutamic acid-rich-like protein 2 | 4 | 0.352040842 | 0.034410103 |
| Q9UK55 | Protein Z-dependent protease inhibitor | 16 | 5.311690898 | 0.035232991 |
| Q07954 | Prolow-density lipoprotein receptor-related protein 1 | 81 | 6.018328267 | 0.035328285 |
| Q9HCN6 | Platelet glycoprotein VI | 6 | 3.574237452 | 0.035521777 |
| A0A075B6H7 | Probable non-functional immunoglobulin kappa variable 3-7 | 2 | 10.91851228 | 0.03567793 |
| Q9C0C9 | (E3-independent) E2 ubiquitin-conjugating enzyme | 18 | 0.408177704 | 0.036058968 |
| P19878 | Neutrophil cytosol factor 2 | 6 | 5.415129345 | 0.036327662 |
| Q6UX06 | Olfactomedin-4 | 5 | 3.90728867 | 0.036438223 |
| Q9UHR4 | Brain-specific angiogenesis inhibitor 1-associated protein 2-like protein 1 | 13 | 10.17498541 | 0.036602889 |
| P78509 | Reelin | 6 | 2.917201534 | 0.036667441 |
| P35813 | Protein phosphatase 1A | 3 | 0.306891375 | 0.036977779 |
| P05164 | Myeloperoxidase | 25 | 3.04753184 | 0.037190069 |
| P48740 | Mannan-binding lectin serine protease 1 | 19 | 2.981674511 | 0.037792869 |
| P98160 | Basement membrane-specific heparan sulfate proteoglycan core protein | 80 | 28.85546744 | 0.038883684 |
| P50225 | Sulfotransferase 1A1 | 2 | 2.015204257 | 0.038999699 |
| P39656 | Dolichyl-diphosphooligosaccharide--protein glycosyltransferase 48 kDa subunit | 7 | 0.340466405 | 0.03902058 |
| Q9C0B5 | Palmitoyltransferase ZDHHC5 | 3 | 2.216858997 | 0.039181659 |
| Q8NI99 | Angiopoietin-related protein 6 | 17 | 4.701397882 | 0.039216769 |
| Q9NXL6 | SID1 transmembrane family member 1 | 3 | 3.232033567 | 0.039228959 |
| Q8TCT9 | Minor histocompatibility antigen H13 | 2 | 0.291260815 | 0.039274382 |
| Q06033 | Inter-alpha-trypsin inhibitor heavy chain H3 | 22 | 7.164073202 | 0.039524168 |
| P09110 | 3-ketoacyl-CoA thiolase, peroxisomal | 8 | 0.437224465 | 0.039873387 |
| Q8WWB7 | Glycosylated lysosomal membrane protein | 2 | 3.05434333 | 0.040049356 |
| P27824 | Calnexin | 16 | 0.41158883 | 0.040166517 |
| Q86SQ4 | Adhesion G-protein coupled receptor G6 | 10 | 4.259434601 | 0.04095106 |
| Q76LX8 | A disintegrin and metalloproteinase with thrombospondin motifs 13 | 3 | 5.079709082 | 0.04111955 |
| O75635 | Serpin B7 | 2 | 0.355428783 | 0.041133961 |
| P15814 | Immunoglobulin lambda-like polypeptide 1 | 2 | 5.127717019 | 0.041189243 |
| O15269 | Serine palmitoyltransferase 1 | 2 | 0.326491605 | 0.041349169 |
| P33121 | Long-chain-fatty-acid--CoA ligase 1 | 14 | 0.45343415 | 0.04195809 |
| P42566 | Epidermal growth factor receptor substrate 15 | 2 | 3.707632924 | 0.042446826 |
| Q9ULP9 | TBC1 domain family member 24 | 3 | 0.494245464 | 0.042950612 |
| P61803 | Dolichyl-diphosphooligosaccharide--protein glycosyltransferase subunit DAD1 | 2 | 0.367311676 | 0.043386834 |
| A0A075B6S5 | Immunoglobulin kappa variable 1-27 | 3 | 13.11858134 | 0.043507354 |
| Q96N66 | Lysophospholipid acyltransferase 7 | 3 | 0.37556564 | 0.043538623 |
| P04844 | Dolichyl-diphosphooligosaccharide--protein glycosyltransferase subunit 2 | 9 | 0.425630815 | 0.043561816 |
| A6NI79 | Coiled-coil domain-containing protein 69 | 2 | 0.391368143 | 0.043754041 |
| O15533 | Tapasin | 4 | 0.421984223 | 0.04416331 |
| A0A0B4J1Y9 | Immunoglobulin heavy variable 3-72 | 6 | 2.345277696 | 0.044611987 |
| Q15555 | Microtubule-associated protein RP/EB family member 2 | 7 | 2.600691797 | 0.046137534 |
| A0A0B4J1U7 | Immunoglobulin heavy variable 6-1 | 2 | 5.683651164 | 0.046889777 |
| P09486 | SPARC | 5 | 3.847393738 | 0.046981814 |
| O00116 | Alkyldihydroxyacetonephosphate synthase, peroxisomal | 7 | 0.222103817 | 0.046985364 |
| P06753 | Tropomyosin alpha-3 chain | 6 | 0.392455149 | 0.047076267 |
| P01714 | Immunoglobulin lambda variable 3-19 | 2 | 4.456226049 | 0.047077355 |
| Q6XQN6 | Nicotinate phosphoribosyltransferase | 7 | 0.292465343 | 0.047958174 |
| Q969H8 | Myeloid-derived growth factor | 3 | 0.485965463 | 0.048460905 |
| P11047 | Laminin subunit gamma-1 | 2 | 2.612602832 | 0.048510273 |
| Q9Y3D6 | Mitochondrial fission 1 protein | 2 | 0.485933705 | 0.049127275 |
| P08183 | ATP-dependent translocase ABCB1 | 9 | 3.302546905 | 0.049272928 |
| A0A0B4J1V0 | Immunoglobulin heavy variable 3-15 | 4 | 5.856864455 | 0.049869121 |

Fold change: mean quantitative values of proteins in EVs of Groups 2 and 3 cases over those of Group 1 cases.

**Supplementary Table 4. Proteins listed in Suppl Table 1 with significantly increased or decreased expression (FDR<0.05) in order of severity in linear regression analysis adjusted for age and sex.**

| Uniprot ID | Protein name | Total Unique Peptide Count | Beta | FDR |
| --- | --- | --- | --- | --- |
| Q13884 | Beta-1-syntrophin | 2 | -114841.8 | 0.006856663 |
| O75367 | Core histone macro-H2A.1 | 4 | 480722.57 | 0.048723727 |

β: regression coefficient, FDR: false discovery rate

**Supplementary Table 5. Proteins listed in Suppl Table 2 with significantly increased or decreased expression (FDR<0.05) in order of severity in linear regression analysis adjusted for age and sex.**

| Uniprot ID | Protein name | Total Unique Peptide Count | Beta | FDR |
| --- | --- | --- | --- | --- |
| Q96KP4 | Cytosolic non-specific dipeptidase | 11 | -2886760.338 | 0.000273022 |
| O95865 | N(G),N(G)-dimethylarginine dimethylaminohydrolase 2 | 5 | -2684408.146 | 0.000434318 |
| O15143 | Actin-related protein 2/3 complex subunit 1B | 11 | -7198822.656 | 0.002078096 |
| Q96FZ7 | Charged multivesicular body protein 6 | 2 | -283285.1207 | 0.002093959 |
| Q8WWA0 | Intelectin-1 | 7 | -31214324.98 | 0.00218064 |
| P48426 | Phosphatidylinositol 5-phosphate 4-kinase type-2 alpha | 5 | -1742775.837 | 0.002590556 |
| O15511 | Actin-related protein 2/3 complex subunit 5 | 4 | -5152136.315 | 0.002757841 |
| P07203 | Glutathione peroxidase 1 | 7 | -3571945.831 | 0.002955461 |
| P15586 | N-acetylglucosamine-6-sulfatase | 2 | -1640960.531 | 0.00300526 |
| O15198 | Mothers against decapentaplegic homolog 9 | 2 | -199087.4566 | 0.003396912 |
| Q14761 | Protein tyrosine phosphatase receptor type C-associated protein | 4 | -3536944.77 | 0.00511592 |
| Q9ULP9 | TBC1 domain family member 24 | 3 | -222362.9988 | 0.006536069 |
| P20963 | T-cell surface glycoprotein CD3 zeta chain | 10 | -3319146.722 | 0.006594908 |
| P21980 | Protein-glutamine gamma-glutamyltransferase 2 | 7 | -2185190.234 | 0.00919872 |
| Q9UJC5 | SH3 domain-binding glutamic acid-rich-like protein 2 | 4 | -979046.3887 | 0.010307066 |
| Q9H3M7 | Thioredoxin-interacting protein | 6 | -675018.4681 | 0.012132653 |
| Q9Y639 | Neuroplastin | 4 | -1518430.832 | 0.012629295 |
| P06753 | Tropomyosin alpha-3 chain | 6 | -8518917.991 | 0.0140626 |
| Q6IAN0 | Dehydrogenase/reductase SDR family member 7B | 7 | 609199.5682 | 0.014354689 |
| P49913 | Cathelicidin antimicrobial peptide | 5 | 6558840.375 | 0.017362253 |
| Q9UBW5 | Bridging integrator 2 | 4 | -490661.0472 | 0.01872093 |
| P02675 | Fibrinogen beta chain | 15 | 22205228.48 | 0.024464035 |
| Q14624 | Inter-alpha-trypsin inhibitor heavy chain H4 | 37 | 1867675635 | 0.028887072 |
| P04003 | C4b-binding protein alpha chain | 27 | 1149488195 | 0.030170149 |
| P46531 | Neurogenic locus notch homolog protein 1 | 25 | 102932220 | 0.030316793 |
| P0DJI8 | Serum amyloid A-1 protein | 4 | 121558707.5 | 0.034614231 |
| P10451 | Osteopontin | 2 | 139985.9099 | 0.036621387 |
| P35813 | Protein phosphatase 1A | 3 | -196694.7016 | 0.037027589 |
| P14543 | Nidogen-1 | 18 | 3324826.549 | 0.038163748 |
| Q9UI12 | V-type proton ATPase subunit H | 5 | -399375.2028 | 0.039388341 |
| O00468 | Agrin | 6 | 811527.4651 | 0.039400794 |
| Q14112 | Nidogen-2 | 6 | 2718366.38 | 0.039407881 |
| P05067 | Amyloid-beta precursor protein | 8 | 735411.5401 | 0.040584285 |
| P01871 | Immunoglobulin heavy constant mu | 20 | 4495072812 | 0.04112882 |
| P22234 | Multifunctional protein ADE2 | 11 | -971181.7739 | 0.042239353 |
| P02776 | Platelet factor 4 | 3 | 2263186010 | 0.042353483 |
| P10643 | Complement component C7 | 34 | 315249488.4 | 0.044456305 |
| P01031 | Complement C5 | 67 | 897471767.5 | 0.044534419 |
| P36959 | GMP reductase 1 | 5 | -567815.5182 | 0.046442482 |
| A6NI79 | Coiled-coil domain-containing protein 69 | 2 | -25993.72021 | 0.047288434 |
| Q9UHC9 | NPC1-like intracellular cholesterol transporter 1 | 9 | 667538.3089 | 0.047978404 |
| O75367 | Core histone macro-H2A.1 | 4 | 480722.5661 | 0.048723727 |

β: regression coefficient, FDR: false discovery rate
